# Phylogenomic analysis of the collagen-like BclA proteins in *Clostridioides difficile*

**DOI:** 10.1128/aem.01582-25

**Published:** 2026-03-03

**Authors:** Francisca Cid-Rojas, Enzo Guerrero-Araya, Christian Brito-Silva, Marjorie Pizarro-Guajardo, César Rodríguez, Daniel Paredes-Sabja

**Affiliations:** 1 Department of Biology, Texas A&M University 14736, College Station, Texas, USA; 2 Millennium Nucleus in the Biology of the Intestinal Microbiota, Millennium Science Initiative Program, ANID 567695, Santiago, Chile; 3 Faculty of Microbiology, CIET, University of Costa Rica 27915, San José, Costa Rica; University of Georgia Center for Food Safety, Griffin, Georgia, USA

**Keywords:** *C. difficile *spores, collagen-like proteins, BclA, *C. difficile *clades, exosporium, pseudogenization

## Abstract

*Clostridioides difficile* is a Gram-positive, anaerobic, spore-forming bacterium and a major nosocomial pathogen, notable for its high genetic diversity. *C. difficile* has been classified into five classical phylogenetic clades (C1–C5) and five cryptic clades (C-I to C-V), reflecting its extensive genome plasticity. In addition, *C. difficile* spores are considered essential for the onset, persistence, and transmission of the disease, and their exosporium layer features hair-like projections formed by the BclA family of proteins (BclA1, BclA2, and BclA3). Previous work in C1 and C2 strains has demonstrated that the absence of these proteins affects spore germination, pathogenesis, persistence, and recurrence of CDI. Nevertheless, the conservation of BclA proteins across different *C. difficile* clades remains unclear. In this work, genomic analysis of more than 25,000 *C. difficile* genomes revealed that the prevalence and variability of BclA are not consistent across classical and cryptic clades. The most represented clade in the data set was C1, where roughly 50% of the genomes possessed all three *bclA* genes. Pseudogenization of *bclA1* and all *bclA* was observed in C2 and C3, respectively. Additionally, the absence of *bclA1* in C4 and both *bclA1* and *bclA3* in C5 further demonstrated the divergence of BclA among *C. difficile* clades. Subsequent analysis revealed high variability in the central collagen-like region (CLR) of all three BclA and a highly conserved *bclA1* pseudogenization event in most members of C2. Restoration of a full-length *bclA1* from strain 630 (C1) into *C. difficile* R20291 (C2) slightly decreased the length of the hair-like projections. Overall, the extensive variability of *bclA*, primarily focused on their CLR, prevalence of a *bclA1* pseudogenization, and absence of *bclA* in specific clades likely impact spore morphogenesis and pathogenesis across the species.

**IMPORTANCE:**

*Clostridioides difficile* is a major cause of healthcare-associated infections, with spores playing a central role in disease transmission, persistence, and recurrence. The outermost spore layer, the exosporium, is critical for interactions with the host and environment, and its structure is shaped in part by the BclA family of proteins. While previous studies have linked BclA proteins to virulence traits, their distribution and conservation across the species’ genetically diverse clades have remained unexplored. This study provides the first comprehensive analysis of *bclA* gene prevalence and variability across more than 25,000 genomes, revealing substantial divergence among classical and cryptic clades. The frequent pseudogenization or absence of *bclA* genes, particularly *bclA1*, and high variability in the collagen-like regions suggest that BclA-mediated exosporium architecture is not universally conserved and may have clade-specific implications for spore morphology and pathogenicity. These findings highlight the need to account for phylogenetic context when studying *C. difficile* spore biology and open new avenues for understanding how structural variation in spores contributes to strain-specific virulence and transmission dynamics.

## INTRODUCTION

*Clostridioides difficile*, a ubiquitous anaerobic spore-producing microbe, is frequently implicated in nosocomial or antimicrobial-associated infections, collectively known as *C. difficile* infections (CDIs) (1). This environmentally persistent pathogen can cause a wide range of symptoms, from mild diarrhea to severe pseudomembranous colitis (2). As a leading cause of healthcare-associated infections, *C. difficile* significantly burdens global health, with incidence rates ranging from 2 to 8 cases per 10,000 patient-days in the United States (3, 4). Conventional treatments for CDI involve antibiotics, such as vancomycin, metronidazole, and fidaxomicin (5). However, despite these interventions, approximately 15%–35% of CDI cases experience recurrence (R-CDI) (6). CDI begins with the germination of endogenous or recently acquired C. *difficile* spores (7), and during colonization, secretes two main virulence factors, toxins TcdA and TcdB, which are responsible for the clinical manifestations of the disease (8, 9).

*C. difficile* spores are the main virulence factor related to disease recurrence (10, 11). During CDI, *C. difficile* begins a sporulation cycle leading to the formation of newly dormant spores (10) that interact with host surfaces (12–15) and persist to cause R-CDI (16). The outermost layer of *C. difficile* spores, called exosporium, possesses surface proteins resembling hair-like projections (11, 17). Transmission electron microscopy (TEM) of spores has revealed that exosporium morphology seems to be strain-dependent, as spores from epidemically relevant strains such as *C. difficile* R20291 have a hair-like nap, whereas *C. difficile* 630, a strain widely used in scientific research, has a compact exosporium layer lacking hair-like structures (11). These hairs resemble those present on spores from members of the *Bacillus cereus* group (*Bacillus anthracis*), which have been determined to be formed by BclA glycoproteins (18, 19). Indeed, Strong et al. (20) identified three *bclA* homologs on the genome of *C. difficile: bclA1, bclA2,* and *bclA3*.

Bacterial proteins like *B. anthracis* BclA and BclB and *Streptococcus pyogenes* Scl1 and Scl2 have been thoroughly characterized previously (18, 19, 21–26). While BclA/B proteins are displayed on the exosporium layer of *B. anthracis* spores, Scl1/2 are displayed on the bacterial cell surface. Notably, both display a central triple-helix rod-shaped structure composed of canonical GXY collagen repeats (27) and a globular-end terminal domain (19, 23, 25, 26); thus, they are referred to as collagen-like proteins. Although *C. difficile* BclA1, BclA2, and BclA3 differ in length, their domain architecture (e.g., N-terminal domain [NTD], collagen-like region [CLR], and C-terminal domain [CTD]) is conserved across the BclA family of proteins (28, 29). It has been shown that hair-like projections formed by the collagen-like BclA3 protein are essential in host-microbe interactions (16). Work in strains 630 (Clade 1) and R20291 (Clade 2) shows their implication in spore-host interactions, colonization, and recurrence of CDI (16, 30). However, their roles and distribution in the wider *C. difficile* taxonomy remain unclear.

With a core genome accounting for only 10%–20% of its pan-genome, *C. difficile* exhibits tremendous genetic diversity (31, 32). This likely contributes to *C. difficile* adaptation to a broad host range and persistence in environmental reservoirs (33) and is reflected by the distribution of the globally known *C. difficile* diversity in five distinct classical clades and five cryptic clades, according to multi-locus sequencing and core-genome phylogenies (31). While Clade 1 is the most represented in public databases, with the broadest geographic distribution (3, 34), Clade 2 includes epidemically relevant strains from ribotype 027 (RT027) commonly implicated in hospital outbreaks (35–37). Clade 3 is uncommon, yet it includes strains sharing phenotypic similarities with Clade 2 strains (38). Clade 4 includes strains that are often clindamycin- and fluoroquinolone-resistant and associated with outbreaks in Asia, North America, and Europe (39). Finally, Clade 5 is the most genetically distant clade from all classical clades, and based on its 96% average nucleotide identity (ANI) to all other four clades, it is hypothesized to be their early predecessor (40). Moreover, several genomospecies, with ANI values lower than 95% to all five classical clades and highly divergent toxin gene architecture, have been identified and classified as cryptic clades (C-I to C-V) (3, 31, 41, 42). Recently, two new classical (C6 and C7) and two new cryptic clades (C-VI and C-VII) have been categorized, with an additional bifurcation of the previously known cryptic clade C-III into C-IIIa and C-IIIb (43).

Despite the wealth of genomic data for *C. difficile*, there is a lack of knowledge on the diversity of spore surface proteins. Therefore, we explored the prevalence and variability of *bclA* genes in more than 25,000 *C. difficile* genomes. Our analysis revealed a high variability of the central collagen-like region of the BclA proteins, indistinctively of the clades to which they belong, and the finding of a conserved *bclA1* pseudogenization event present across most members of C2 and C3, which was not observed for *bclA2* or *bclA3*. Complementation of *C. difficile* R20291 (C2) with a full-length *bclA1* from strain 630 (C1) decreased the length of hair-like projections. Overall, the extensive variability of *bclA*, primarily on its CLR, prevalence of a *bclA1* pseudogenization, and absence of *bclA* in specific clades likely impact spore morphogenesis and pathogenesis across the species. Hence, this work could offer insights into developing therapeutics aimed at broad-spectrum or clade-specific strategies to combat *C. difficile* infections.

## MATERIALS AND METHODS

### 3D structural inference

AlphaFold3 (AF3) (44), a state-of-the-art protein folding prediction algorithm, was used to determine the three-dimensional structure of the proteins under investigation. Predictions were performed with 24 iterative cycles (seeds), and the highest-ranking predicted structure (rank 0) with the lowest predicted alignment error (PAE) was selected for downstream analyses.

### Genomic data set

From a recent parallel study that used publicly available genomes from Enterobase (https://enterobase.warwick.ac.uk), genomes with an *N*_50_ > 5,000 bp were considered, leading to a total of 25,165 draft assembly *C. difficile* genomes (43). All assembled genomes underwent multi-locus sequence typing utilizing FastMLST version 0.0.15 (45).

### Core genome and phylogeny

Core-genome identification was performed using DIAMOND blastp (46) with a bidirectional best hit approach, using *C. difficile* 630 as reference (GenBank AM180355.1). Protein-coding genes were predicted using Prodigal, a widely used and accurate gene prediction tool designed for microbial genomes (47). Genes present in at least 99% of isolates were considered core genes. Each core gene was aligned individually using MAFFT. Given the large number of isolates in the data set, the focus was set on single-nucleotide polymorphisms (SNPs) present in the alignment for constructing the core-genome phylogenetic tree. To achieve this, RapidNJ was employed (https://github.com/somme89/rapidNJ), a fast and efficient algorithm for constructing phylogenetic trees based on the Neighbor-Joining method. The tree incorporates *Clostridium mangenotii* as an outgroup to root the tree and provide context.

### Extraction and distribution of *bclA* ORFs

To extract the sequences of each BclA, open reading frames (ORFs) were predicted in each genome of the data set using Prodigal (47). Following the prediction of ORFs, the genomic region of interest was defined by searching the up- and downstream gene flanking each *bclA* using DIAMOND (46). Subsequently, these *bclA*-containing regions were extracted for further investigation and analysis. To ensure accuracy and precision in the extraction process, this final step was performed manually. To minimize redundancy in the data set and improve computational efficiency, deduplication using CD-HIT was performed, a widely utilized clustering tool that identifies and removes duplicated sequences based on sequence similarity (48). Clustering was performed using a 100% identity threshold and 100% coverage, ensuring that only completely identical sequences were grouped together. This step ensured that the data set contained only unique sequences, enabling more accurate and meaningful downstream analyses. However, it should be noted that the genomic data set used in this work was filtered by a *N*_50_ > 5 kb threshold in Guerrero-Araya et al. (43), which may be relatively permissive, particularly for repetitive loci of ~2 kb (*bclA* gene sizes), which could lead to artifactual pseudogenization. Hence, to minimize this artifact-pseudogene generation, the following criteria were included in the pipeline: (i) the loci of interest were localized using conserved flanking regions, followed by manual inspection and curation of the segments; (ii) true pseudogenes from low-quality or fragmented assemblies (e.g., sequences with Ns, contig breaks, and obvious truncations due to missing sequence) were classified as “containing Ns” or “interrupted” and not considered as pseudogenes. By contrast, a pseudogene was defined when a clear nonsense mutation or frameshift was present within an otherwise intact locus. Finally, custom Python scripts were used to generate schematic representations of the results. These visualizations provided a clear and concise representation of the findings, facilitating the interpretation and communication of the insights gleaned from the study.

### Sequence alignment

Clustal Omega (49), a widely recognized and efficient tool for generating multiple sequence alignments, was used to align the sequences of interest. EMBOSS Needle was used to generate pairwise optimal global alignments using the Needleman-Wunsch algorithm. Both software packages were run using default parameters.

### Bacterial strains and growth conditions

*C. difficile* strains (Table 1) (50) were routinely grown at 37°C under anaerobic conditions in a Coy Vinyl Anaerobic chamber (COY Laboratory Products, USA) in BHIS medium: 3.7% (wt/vol) brain heart infusion (BD, USA) supplemented with 0.5% (wt/vol) yeast extract (BD, USA) and 0.1% (wt/vol) L-cysteine (Thermo Fisher Scientific, USA). When needed, BHIS medium was supplemented with 15 µg mL^−1^ thiamphenicol (Sigma-Aldrich, USA), 16 µg mL^−1^ cefoxitin (Sigma-Aldrich, USA), and 250 µg mL^−1^ D-cycloserine (Chem-Impex International, USA). *Escherichia coli* strains (Table 1) were routinely grown aerobically at 37°C with shaking at 180 rpm in Luria-Bertani (LB) medium: 1% (wt/vol) trypticase, 0.5% (wt/vol) yeast extract (BD, USA), and 1% (wt/vol) NaCl (VWR, USA). When needed, LB medium was supplemented with 50 µg mL^−1^ chloramphenicol (Chem-Impex International, USA). To grow strains in plates, 1.5% (wt/vol) agar (VWR, USA) was added to BHIS or LB medium.

**TABLE 1:** Bacterial strains and plasmids used

Strain or plasmid	Relevant characteristics	Source/reference
*C. difficile* strains		
630	Ribotype 012	(51)
R20291	Ribotype 027, epidemically relevant strain	(52)
R20291 *∆pyrE*	R20291 isogenic *pyrE* mutant	(53)
R20291 *∆pyrE::pyrE*	R20291 isogenic *pyrE* mutant complemented with wild-type *pyrE* into the *pyrE* locus	(16)
R20291 *∆pyrE::pyrE_bclA1_630_*	R20291 isogenic *pyrE* mutant complemented with wild-type *pyrE,* promoter and ORF of *bclA1*_630_ strain into the *pyrE* locus	This project
*E. coli* strains		
NEB Turbo	EcoKr^–^m^–^, McrBC^–^	NEB
CA434	*hsd20*(r^B^-, m^B^-, *recA13*, *rpsL20*, *leu*, *proA2*) with IncPβ conjugative plasmid R702	(54, 55)
Plasmids		
pMTL-YN2C	Pseudo suicide vector containing *Clostridium perfringens catP* cassette and *Clostridium sporogenes pyrE*; carries unaltered *colE1 E. coli* replicon; *traJ* encoding transfer function of the RP4 oriT region; RepA and Orf2, replication region of *Clostridium botulinum* plasmid pBP1; a left-hand homology arm encompassing a 300 bp internal fragment of the R20291 *pyrE* gene, lacking 50 nucleotides from 5′-end and 235 bp from 3′-end; a right-hand homology arm comprising 1,200 bp region of DNA immediately downstream *pyrE*; additional DNA segments inserted between left- and right-hand homology arms that carry a copy of *lacZ*′ containing a multiple cloning site region and a transcriptional terminator of the ferredoxin gene	(53)
pCBS1	A 2,759 bp BamHI-EcoRI fragment containing the full gene sequence of CD630_03320 (*bclA1*) of the 630 strain cloned into the BamHI-EcoRI site of pMTL-YN2C	This project

### Plasmid construction

To complement *bclA1_630_* in *C. difficile* R20291*,* a PCR fragment of 2,759 bp was amplified from *C. difficile* 630 (CD630_03320) using primers FP-*bclA1*-CD630-EcoRI and RP-*bclA1*-CD630-BamHI (Table 2). The fragment containing the complete *bclA1* ORF plus 677 bp upstream of the start codon was cloned into the BamHI-EcoRI sites of pMTL-YN2C (53), resulting in plasmid pCBS1 (Table 1). Plasmid was sequenced (Plasmidsaurus, USA) to verify correct construction before downstream use. SnapGene version 5.0.8 was used to analyze Oxford Nanopore plasmid sequencing files (Plasmidsaurus, USA). A consensus circular sequence (.gbk) was aligned to *in silico*-constructed pCBS1 plasmid using the default SnapGene aligner. No sequence discrepancy was identified in alignment.

**TABLE 2:** Primers used

Name	Sequence (5′–3′)^*a*^
FP-*bclA1*-CD630-EcoRI	*TTTTTT*GAATTCAATTTATATAAAAACTAGATTTAGTTAT AAATAAAAATTAAAAATATATA
RP-*bclA1*-CD630-BamHI	*AAAAAA*GGATCCTTAAGTCATAACAGTATCAGCTATTC
FP-235*pyrE*	GGTGGAGAAGTTGTAGGTGTTGCATG
RP 0190	GGGAGGATGGTTCTGGAACCAG

^*a*^ Restriction sites are underlined, and added sequences for proper restriction digestion are in italics.

### Complementation by allelic exchange at the *pyrE* locus

For the complementation of *bclA1_630_* into *C. difficile* R20291 *∆pyrE* strain (53), pCBS1 was transformed into *E. coli* CA434 and subsequently conjugated into *C. difficile* (53). Briefly, transconjugants were selected in defined *C. difficile* minimal medium solidified with 1.5% (wt/vol) agar (56). Plasmid excision confirmation was made by negative selection on BHIS thiamphenicol plates, and sensitive clones were further screened by PCR using primers FP-235*pyrE* and RP 0190 (Table 2) to detect complementation under the *pyrE* locus. Complemented strains were whole-genome sequenced (SeqCenter, USA) to confirm their genetic background and that no additional SNPs were introduced during genetic manipulation.

To confirm the proper construction of the mutant strain, Geneious Prime 2025.1.3 was used to analyze whole-genome sequencing (SeqCenter, USA) Illumina data. Reads were mapped to *in silico*-constructed *C. difficile ∆pyrE::pyrE_bclA1_630_* genome (derived from CP029423) using Bowtie2 version 7.2.2, with default parameters. Whole genome variant calling was performed by Geneious variant finder with a minimum coverage of five reads and a minimum variant frequency of 0.95; no additional single-nucleotide variations were identified in comparison to the parental strain.

### Growth assays

*C. difficile* strains were cultured anaerobically on pre-reduced BHIS broth supplemented with 0.5% glucose (BHISG) and 0.1% sodium taurocholate (Sigma, USA) for 16 h at 37°C to prevent sporulation. Overnight culture was then diluted 1:3 with pre-warmed BHISG broth supplemented with 0.1% sodium taurocholate (Sigma, USA) and incubated for 2 h at 37°C to allow the culture to enter the exponential phase. Subsequently, it was diluted 1:100 in fresh BHIS, and 100 μL was added to the wells of a 96-well plate. The OD_600_ was measured every 3 min during 22 h at 37°C using a Cerillo’s Alto microplate reader. Three independent biological replicates, each with four technical replicates, were performed for each sample.

### Spore purification

As previously described (57), BHIS broth supplemented with 0.1% taurocholate and 0.2% fructose was inoculated with a single *C. difficile* colony and grown anaerobically at 37°C for 16 h. Overnight culture was diluted to 1/3 in prewarmed BHIS supplemented with 0.1% taurocholate and 0.2% fructose and incubated at 37°C for 2 h. An aliquot of 250 µL was plated on pre-reduced 70:30 agar plates (35 mL): 6.3% (wt/vol) peptone (BD, USA), 0.35% (wt/vol) protease peptone (BD, USA), 0.07% (wt/vol) ammonium sulfate (NH_4_)_2_SO_4_ (Merck USA), 0.106% (wt/vol) Tris base (Omnipur, Germany), 1.11% (wt/vol) brain heart infusion extract (BD, USA), 0.15% (wt/vol) yeast extract (BD, USA), and 1.5% (wt/vol) agar (VWR, USA). Plates were incubated for 5 days at 37°C under anaerobic conditions in a Vinyl Anaerobic chamber (COY Laboratory Products, USA). Plates were removed from the chamber, and growth was scraped into a microcentrifuge tube with 1 mL ice-cold sterile Milli-Q water. The cells were resuspended and placed overnight at 4°C. Next, the sporulated culture underwent five washes with ice-cold Milli-Q water in a microcentrifuge at 18,400 × *g* for 5 min each. Spores were purified by density using 45% (wt/vol) autoclaved Nycodenz (Axell USA) solution and centrifuged at 18,400 × *g* for 20 min. Bacterial debris was removed, and the spore pellet was separated and washed five times at 18,400 × *g* for 5 min with ice-cold sterile Milli-Q water to remove Nycodenz. Spores were counted in a Neubauer chamber, and the suspensions were adjusted to contain 5 × 10^9^ spores mL^−1^ and stored at −80°C until use.

### Biofilm formation

Biofilm formation was measured as described with modifications (58). Briefly, BHISG broth was inoculated with a single *C. difficile* colony and grown anaerobically at 37°C for 16 h. Overnight culture was diluted 1:3 with pre-warmed BHISG broth and incubated for 4 h at 37°C. Next, cultures were diluted 1:50 in BHISG, and 1 mL aliquots were deposited in 24-well tissue culture-treated plates, incubated at 37°C in anaerobic conditions for 24 h, and then removed from the anaerobic chamber to carry out the following steps at room temperature. Biofilm biomass was gently washed twice with sterile 1× phosphate-buffered saline (PBS), left to dry overnight, and subsequently stained with 750 μL of crystal violet (CV, 0.1% [wt/vol]) for 1 h. CV was removed by inversion, gently washed twice with 1× PBS, and left to air dry. All washing steps were performed by inversion. CV bound to biofilm was extracted by incubating for 2 h at room temperature with 1 mL of 100% ethanol, and the absorbance was measured at OD_570_ with a plate reader (BioTek, Synergy H1). The solubilized CV was diluted 1:2 and 1:4 to keep the reading within the linear range of the spectrophotometer.

### Sporulation assay by ethanol resistance and phase-contrast microscopy

*C. difficile* sporulation efficiency was assayed as previously described, with some modifications (59). BHIS inoculation and 70:30 plating were performed as previously described in spore purification methods. After 24 h, half of each sample plate was harvested into 1 mL of 1× PBS, OD_600_ was measured, and the absorbance was adjusted to 1. From this solution, a 250 μL aliquot was treated with 100 µL (treated) of 100% ethanol or 1× PBS (untreated; control) for 15 min at room temperature, serially diluted in 1× PBS from 10^−1^ to 10^−7^, and spot plated 5 µL in triplicate onto BHIS + 0.1% sodium taurocholate plates. After 24 h at 37°C under anaerobic conditions, the sporulation frequency was calculated as the proportion of spores that germinated after ethanol treatment divided by the total number of CFU in the untreated sample. The ratio of vegetative cells versus spores/sporulating cells was also determined using phase contrast microscopy (Leica DMRX; Germany).

### Transmission electron microscopy

Spore pellets (2 × 10^8^) were fixed with 3% glutaraldehyde in 0.1 M cacodylate buffer (pH 7.2), incubated overnight at 4°C, and subjected to a second fixation step with 1% Osmium tetroxide in 0.1 M cacodylate buffer for 2 h at room temperature. Pellets were washed and stained for 30 min with 1% tannic acid and subsequently embedded in Spurr resin (60, 61). Thin sections (90 nm) were obtained using a microtome, placed on glow-discharge carbon-coated grids, and double-stained with 2% uranyl acetate followed by lead citrate. Spores were analyzed using a Philips Tecnai 12 Bio Twin microscope at the Unidad de Microscopía Avanzada, Pontificia Universidad Católica de Chile.

To analyze spore layer thickness and the length of hair-like projections, transmission electron micrographs of representative spores, exhibiting either thin or thick exosporium, were selected as previously described (61–63). For each spore, measurements were performed at three distinct locations with ImageJ (1.51m9), and the mean value was represented in the graphs, as previously described (64).

### Quantification of appendage by phase-contrast microscopy

Purified spore suspensions were analyzed by phase contrast microscopy for the presence of a protrusion of ~30% of the length of the spore, known as the polar appendage. This structure is more frequent in *C. difficile* 630 than in strain R20291 (61, 65). At least 500 spores per batch were counted from three independent spore preparations.

### Statistical analyses

Statistical significance was determined using GraphPad Prism version 9.0, and the exact analysis of each experiment is described in the figure legends.

## RESULTS

### Structural modeling of BclA proteins of strains 630 and R20291

**Fig 1:**
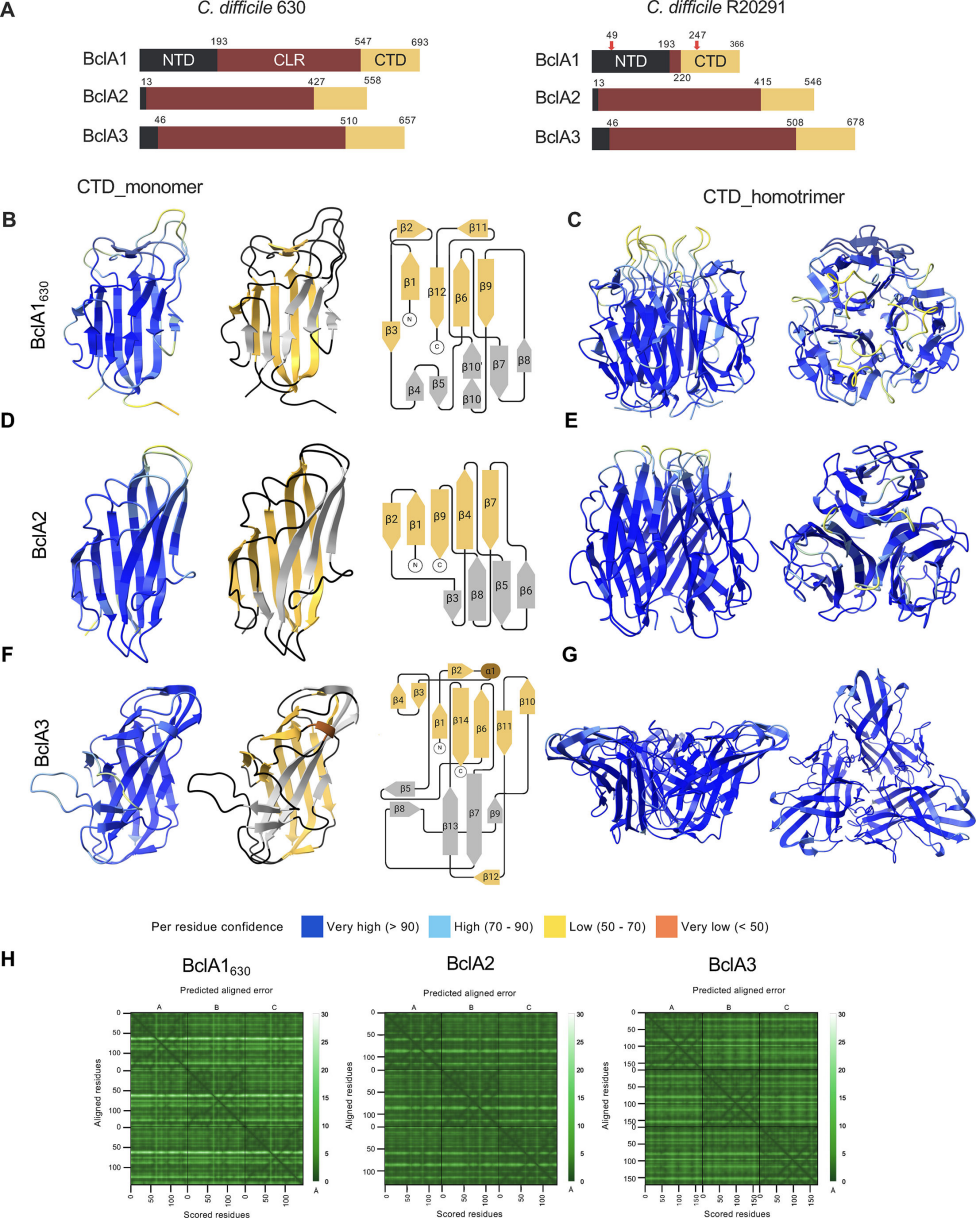
BclA domain structure and AlphaFold3 predictions of *C. difficile* 630 and R20291 CTD. (**A**) Schematic representation of *C. difficile* 630 and R20291 BclA1, BclA2, and BclA3 protein sequences. NTD (black), N-terminal domain; CLR (maroon), collagen-like region; and CTD (yellow), C-terminal domain. In red arrows are depicted two stop codons that lead to early termination of translation of BclA1_R20291_. (**B, D, and F**) AlphaFold3 prediction of CTD monomers of BclA1_630_, BclA2_R20291_, and BclA3_R20291_. Topology models are shown to the right. *α*-helix and loops are displayed in brown and black, respectively. Yellow *β*-sheets are located on the inner face of homotrimer models. Gray *β*-strands are located on the outer face of homotrimer models. Encircled “N” and “C” represent the amino- and carboxyl-terminus, respectively. Structural predictions indicate that BclA1_630_ had a total of 12 *β*-sheets, BclA2_R20291_ had a total of 9 *β*-sheets, and BclA3_R20291_ encodes 14 *β*-sheets and 1 *α*-helix. (**C, E, and G**) AlphaFold3 prediction of CTD homotrimers of BclA1_630_, BclA2_R20291_, and BclA3_R20291_, and in (**H**) their PAE is shown.

*C. difficile* encodes at least three orthologs of the BclA family of collagen-like proteins (66) (Fig. 1A; Fig. S1). Amino acid sequence comparison reveals that BclA1 of strains 630 (Ribotype 012, clade C1) and R20291 (Ribotype 027, clade C2) share ~51% identity (Fig. S2). However, the identity increases in the case of BclA2 with ~95% (Fig. S3) and in a lesser amount in BclA3 with ~87% identity (Fig. S3). A particular characteristic of *bclA1* is its pseudogenization in R20291 by two nonsense mutations at (i) nucleotide position A145T, resulting in an early-stop codon that leads to the formation of a small 48 amino acid NTD polypeptide; and (ii) nucleotide position C739T, which yields an additional stop codon (Fig. 1A; Fig. S1A). Despite these nonsense mutations, the downstream DNA sequence in *bclA1*_R20291_ follows the same structure as a canonical *bclA1* (Fig. S1A). When the comparison is restricted to the NTD and CTD of *bclA1*, identity reaches 97% (Table S1). The same is observed for BclA2 and BclA3 when the alignment was restricted to individual domains, where gap-free alignment regions revealed more than 94% identity, except for CLR of BclA3, which reached only 77% of identity (Table S1). For a better comparison between BclA1 from strains 630 and R20291 at a protein level, the two nonsense mutations were repaired to obtain a restored full-length (fl) BclA1_R20291_ sequence termed rep-BclA1_R20291_. Overall, BclA proteins of these two strains belonging to different clades were similar, but the CLR showed the least similarity between the domains.

Given the differences in BclA1 and similarities in BclA2 and BclA3 between strains 630 and R20291, AF3 was used to create structural predictions of full-length BclA1_630_, rep-BclA1_R20291_, BclA2_R20291_, and BclA3_R20291_. Initial monomeric predictions for all fl-BclA showed low-confidence models for almost the entire protein (Fig. S5A, B, S6A, and S7A). Upon analyzing the individual domains, the CLR of all four proteins proved to be disorganized with very low predicted confidence. However, the CTDs of BclA2 and BclA3 were confidently predicted as globular structures with low expected positional error (Fig. S6A and S7A). Interestingly, the NTDs of BclA1_630_ and rep-BclA1_R20291_ were predicted as globular domains, while the NTDs of BclA2 and BclA3 were not assigned a secondary structure (Fig. S5A, B, S6A, and S7A). Among these NTDs, rep-BclA1_R20291_was predicted with higher confidence than that of BclA1_630_, likely because the longer disorganized CLR domain in BclA1_630_ could be affecting AF3 predictions (Fig. S5A and B). Overall, BclA monomers had a reliable CTD model, while failing to accurately predict NTD and CLR.

Mammalian collagen proteins have a globular trimerization domain at one of their terminal domains, which, together with their intrinsic GXY repeats, is a driving force for the formation of triple-helix structures (67, 68), suggesting that BclA are likely to form stable, predictable trimers due to the same protein domain organization. Strikingly, although homotrimers were predicted for all fl-BclA, the confidence of these interactions was limited mainly to their CTDs. Both BclA1_630_ and rep-BclA1_R20291_ exhibited an apparent low confidence homotrimerization prediction (Fig. S5C and D). By contrast, BclA2 had the highest predicted confidence, forming a tight CTD-protein complex (Fig. S6B), followed by BclA3 (Fig. S7B). Unfortunately, AF3 could not predict the triple-helical structure in *C. difficile* collagen-like BclA proteins.

It has been previously shown that the smallest collagen-like proteins known to self-trimerize are synthetic collagen mimetic peptides with at least six GXY repeats (69, 70). Therefore, a truncated CLR version with 10 GXY repeats, containing the first and last 5 repeats of the collagen-like region, was created for each BclA to evaluate their predictability using AF3. The structures of 10 GXY BclA improved the structural prediction. AF3 models of these versions for BclA2 and BclA3 revealed a more accurate organization of BclA trimers (Fig. S8B and C). However, although prediction improved for the truncated compared to full-length BclA1, trimerization of the CTD was not as confident as for truncated versions of BclA2 and BclA3 (Fig. S8). AF3 has limitations in predicting nonglobular proteins (71), which explains the poor predictions observed for CLR and NTD of full-length and truncated BclA version (Fig. S5 to S8). By contrast, AF3 confidently predicted homotrimerization of the C-terminal domains in full-length and truncated versions of BclA (Fig. S6B, S7B, and S8), supporting the hypothesis that all three BclA form homotrimers.

### Structural modeling of BclA heterotrimer proteins

Collagen molecules can bind to different types of collagens, forming heterotrimeric proteins (67, 72, 73). Therefore, to test the hypothesis that *C. difficile* BclA orthologs could form heterotrimers, AF3 was employed to predict the structure of trimeric complexes composed of different BclA proteins. For these models, only the CTD domain was used due to its high-confidence predicted structure. A combination of different BclA CTD sequences was used as separate chains, allowing AF3 to model potential inter-chain interactions and assess compatibility in forming stable trimeric assemblies. Resulting predictions revealed no expected interactions between the CTD of different BclA proteins (CTD_BclA2-BclA3_R20291_ [Fig. S9] and CTD_BclA1-BclA2-BclA3_630_ [Fig. S10]), even though most molecules were predicted with high confidence as independent units. These results support the notion that *C. difficile* BclA do not form heterotrimeric protein complexes.

Further analysis of CTDs demonstrated that BclA1_630_, BclA2, and BclA3 are formed by a total of 12, 9, and 14 predicted beta sheets, respectively, and only the CTD of BclA3 had an alpha helix between beta sheets 2 and 3 (Fig. 1B, D, and F). While they all form a homotrimer barrel-type structure predicted with high confidence, BclA1_630_ and BclA2 have a ball-like shape (Fig. 1C and E), and BclA3 has a trapezoid-like shape that, when viewed from above, forms an isosceles triangle (Fig. 1G). Interestingly, the areas of lowest confidence in the inference of the three-dimensional structure are in the coils at the tip of each monomer conforming the structures (Fig. 1C, E and G). These structural differences suggest that *C. difficile* BclA1, BclA2, and BclA3 may have different functions in exosporium structure and roles in spore-host interactions.

### Structural similarities between *C. difficile* and *B. anthracis* BclA proteins

**Fig 2:**
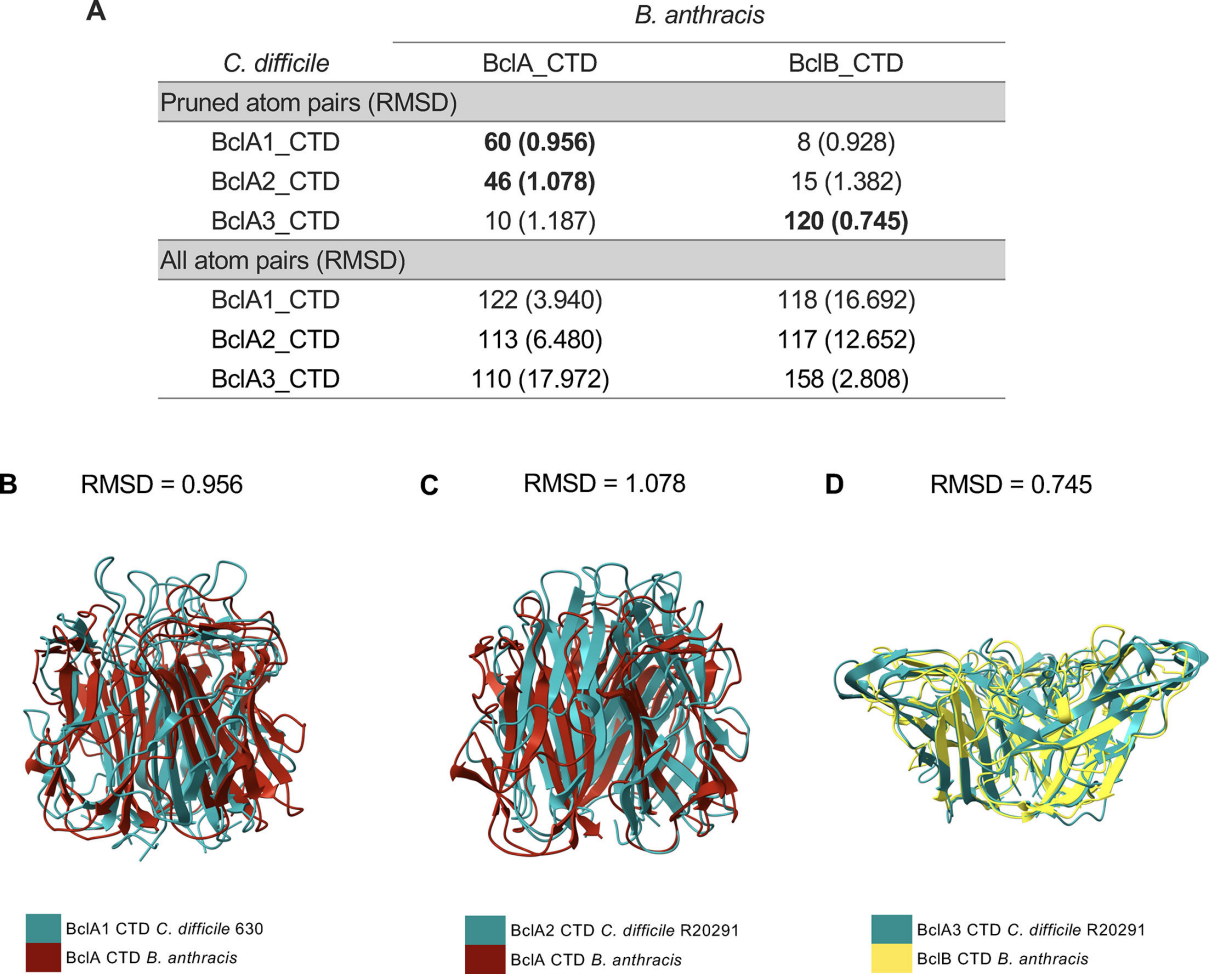
*C. difficile* BclA comparison with BclA and BclB CTD domain from *B. anthracis*. AlphaFold 3 predicted CTD homotrimer structures of *C. difficile* (Cd) and *B. anthracis* (Ba) were superimposed on ChimeraX (version 1.9) using the “matchmaker” command with default parameters. (**A**) RMSD for both pruned and all atom pairs is reported for all combinations possible. Numbers in bold represent the lowest RMSD achieved in each protein comparison. Superimposed pairs are (**B**) Cd_BclA1_CTD_630_ with Ba_BclA_CTD, (**C**) Cd_BclA2_CTD_R20291_ with Ba_BclA_CTD, and (**D**) Cd_BclA3_CTD_R20291_ with Ba_BclB_CTD. The genomes of *C. difficile* 630 (AM180355.1), *C. difficile* R20291 (CP029423), and *B. anthracis* (AE016879.1) were used as references.

To investigate how the predicted structures of *C. difficile* collagen-like proteins compare to those of the well-studied *B. anthracis* BclA and BclB, AF3 was used to model CTD homotrimers. *C. difficile* BclA CTD domains were independently superimposed to the CTD of *B. anthracis* BclA and BclB using ChimeraX (Fig. 2A). To quantify atomic differences between the superimposed proteins, root mean square deviation (RMSD) was calculated. Both BclA1_630_ and BclA2 _R20291_ CTD had the best hit with the CTD of *B. anthracis* BclA, with an RMSD of 0.956 and 1.078, respectively (Fig. 2B and C). On the other hand, BclA3 _R20291_ CTD showed a high structural similarity with *B. anthracis* BclB CTD, with an RMSD of 0.745 (Fig. 2D), yet low amino acid sequence identity (15.8%–19.5%) was observed between these domains (Fig. S11). These results suggest that the CTDs of both species share comparable predicted structures despite their low level of amino acid identity.

### Phylogenomic distribution of BclA across *C. difficile* lineages

**Fig 3:**
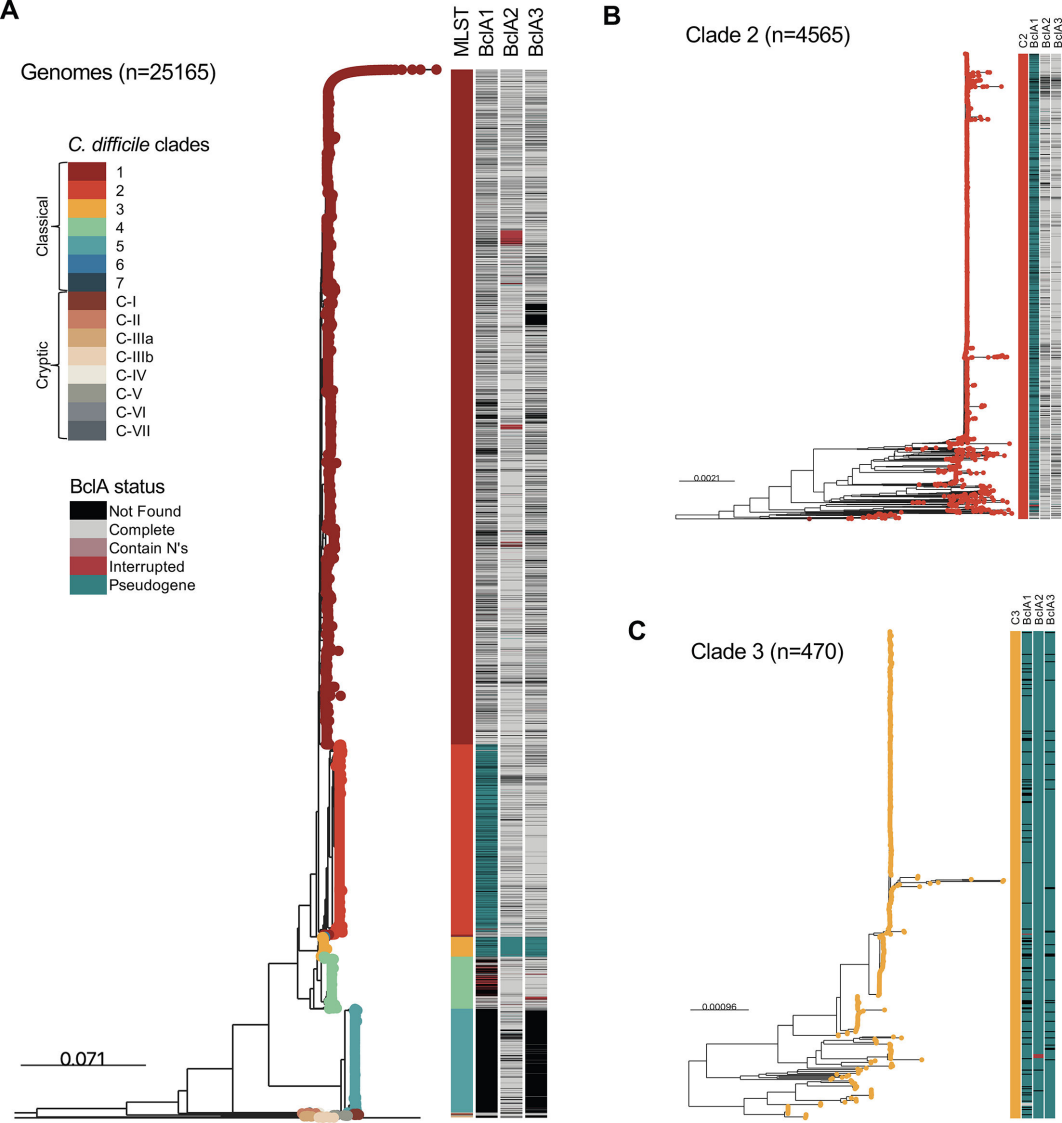
Distribution of BclA in the known *C. difficile* diversity. (**A**) Phylogenetic tree of 25,000 core genomes of *C. difficile*, showing the clade each genome belongs to. Horizontal bars represent the state of BclA in a particular genome (not found, complete, containing Ns, interrupted, or pseudogenized). (**B**) Zoomed-in view of the clade C2 subtree. (**C**) Zoomed-in view of the clade C3 subtree.

To further explore the diversity of BclA in *C. difficile*, a set of 25,165 genomes was analyzed (Table S2). The presence (e.g., intact ORF or pseudogenized) of *bclA1* was found in 15,680 genomes (Table S3), *bclA2* in 21,540 genomes (Table S4), and *bclA3* in 17,685 genomes (Table S5). The distribution of hits in a phylogenetic core-genome tree is shown in Fig. 3A. In the case of *bclA1* occurrence, a homogeneous distribution is observed in all clades except for clade C4, where *bclA1* was absent in most (74%), whereas in clade C5, *bclA1* was completely missing (Fig. 3A; Fig. S12A and B; Table S6). *bclA2* and *bclA3* were ubiquitous across all clades, except for clade C5, where *bclA3* was absent in 97.6% of the genomes (Fig. 3A; Fig. S12B; Table S6).

Clade C1 comprised 16,254 genomes, among which the majority harbored full-length ORFs, encoding at least one *bclA* ortholog: *bclA1* in 68.6%, *bclA2* in 86.0%, and *bclA3* in 74.2% of genomes. Notably, 54.7% (*n* = 8,889) of genomes in this clade contained all three *bclA* homologs. A smaller subset presented only *bclA2* (10.3%), *bclA3* (3.2%), or *bclA1* (1.5%) individually. Pseudogenized *bclA* genes are rare in this clade, with *bclA1*, *bclA2*, and *bclA3* pseudogenization observed in only 0.1%, 0.2%, and 0.2% of genomes, respectively. These results indicate that clade C1 harbors a largely intact and widespread distribution of *bclA*, with minimal gene disruption (Fig. 3A; Tables S6 to S8).

Of the 4,565 genomes analyzed in clade C2, only 56 genomes (1.2%) contained a complete *bclA1* ORF. In contrast, 80.9% (*n* = 3,694) exhibited a pseudogenized *bclA1*, indicating a clade-wide disruption event for this gene. Despite this, most genomes (69.3%) retained all three *bclA* homologs, including pseudogenized copies. A smaller fraction harbored only one *bclA* ORF (*bclA1*, 2.9%; *bclA2*, 3.5%; and *bclA3*, 2.8%), and pseudogenized *bclA2* and bclA3 were found in 0.2% and 0.1% of genomes, respectively. These findings suggest that while *bclA1* is frequently disrupted in clade C2, the overall *bclA* repertoire remains largely preserved (Fig. 3B; Tables S6 to S8).

Among the 470 genomes analyzed from clade C3, pseudogenization was widespread, with 81.7% of genomes containing at least one disrupted *bclA* gene. Complete ORFs for *bclA2* and *bclA3* were absent from all genomes, while only three genomes carried a complete *bclA1* (0.6%). Pseudogenized alleles were prevalent: *bclA1* in 84.0%, *bclA2* in 98.7%, and *bclA3* in 93.8% of genomes. Only a small subset contained a single *bclA* gene (*bclA2*, 4.3%, *n* = 20; *bclA3*, 0.2%, *n* = 1), and no genome harbored only *bclA1*. This suggests that clade C3 might not have functional BclA proteins, based on their highly pseudogenized alleles (Fig. 3C; Tables S6 to S8).

There were 1,254 genomes available for clade C4. Most of them showed full-length ORFs for *bclA2* (90.6%) and *bclA3* (82.6%), while *bclA1* was found intact in only 25.5% of genomes. However, only 16.9% (*n* = 212) of the genomes contained all three full-length *bclA* homologs. A minority of genomes included only *bclA1* (0.9%), *bclA2* (6.9%), or *bclA3* (4.6%), and pseudogenization was limited, occurring in 0.3% (*bclA1*), 0% (*bclA2*), and 0.16% (*bclA3*) of genomes. These data show that while *bclA2* and *bclA3* are well conserved in Clade 4, *bclA1* is frequently absent or incomplete, limiting the presence of the full set of paralogs (Fig. S12A; Tables S6 to S8).

Out of the 2,491 genomes of Clade 5 analyzed, 79.7% possessed a complete *bclA2* ORF. Interestingly, the majority were missing *bclA1* (100%) or *bclA3* (97.6%), and some genomes (19.6%) were missing all three *bclA*. Pseudogenized *bclA2* and *bclA3* alleles were present in 0.4% and 0.04%, respectively. Overall, these results suggest that Clade 5 genomes only have BclA2 in their repertoire (Fig. S12B; Tables S6 to S8). Unfortunately, the *C. difficile* data set had few representatives of recently described Clade 6 (*n* = 5) and Clade 7 (*n* = 1) (43); therefore, it was not possible to determine statistical trends for them (Table S6).

Regarding the cryptic clades, almost all genomes of the recently discovered clades C-IIIa and C-IIIb lacked *bclA1* and *bclA3*, while in roughly 50%, *bclA2* was missing. Meanwhile, *bclA2* was absent in all C-II and C-IV genomes and missing in 57% of clade C-V (Fig. S12C; Tables S6, S7, and S9). Altogether, these results suggest that the extensive *bclA* variability observed across clades could lead to distinct functions in virulence-associated traits associated with spore-host interactions.

### Pseudogenization of *bclA* across *C. difficile* clades

**Fig 4:**
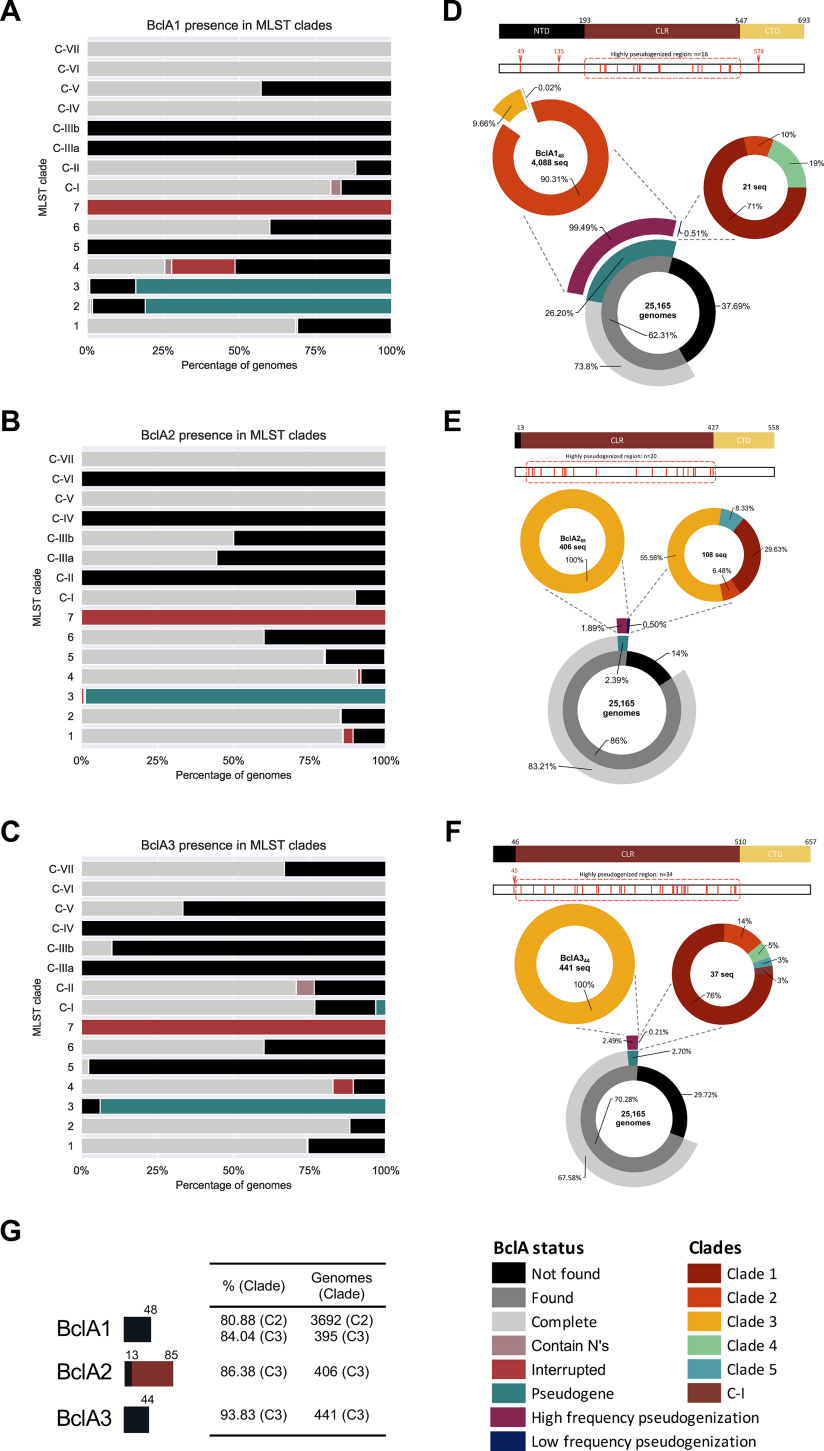
State of BclA in the known diversity of *C. difficile*. Prevalence of found conditions of (**A**) BclA1, (**B**) BclA2, and (**C**) BclA3 on each clade. Prevalence of pseudogenized (**D**) BclA1, (**E**) BclA2, and (**F**) BclA3 sequences in each *C. difficile* clade. Schematic representation of BclA protein sequences is depicted on the top of each donut chart. Truncated BclA sequences were manually curated to locate the position of early stop codons, represented as red lines in a white rectangle below the protein schematic. (**G**) High abundant BclA truncated protein types. CLR (maroon), collagen-like region; CTD (yellow), C-terminal domain; and NTD (black), N-terminal domain. In this sub-data set, only clades represented in the figure are present in the legend.

As stated before, pseudogenization of *bclA1* was seen in 80.9% of clade C2 and 84% of clade C3 genomes (Fig. 4A and G; Tables S6 and S8), while pseudogenized *bclA2* and *bclA3* were observed in 98.7% and 93.8% of clade C3 genomes, respectively (Fig. 4B, C, and G; Tables S6 and S8). Notably, specific pseudogenized events were highly abundant for each of the *bclA*. At the amino acid sequence level, *bclA1* pseudogenization occurred at residue 49 in 99.5% of genomes, consistent with the pattern observed in clades C2 and C3 (Fig. 4D and G; Table S10). In the case of *bclA2*, pseudogenization events occurred at residue 86 in 79% of cases, all of them observed in clade C3 genomes. This *bclA2* allele yielded a truncated protein that retained its NTD and the first 72 (17.9%, 72/402) residues of its CLR (Fig. 4E and G; Table S11). For *bclA3*, pseudogenization events occurred at residue 44, resulting in predicted proteins that retain nearly the full NTD (44/46 residues), all of them occurring in clade C3 (Fig. 4F and G; Table S12). These intermediate-length truncations in BclA suggest the potential for limited structural function, such as the formation of short hair-like exosporium projections. Together, these findings indicate that clade C3 has undergone extensive and consistent *bclA* pseudogenization, likely altering spore surface architecture while preserving partial domain structures in some homologs.

Low-abundant and unique *bclA* pseudogenes were also studied. These alleles were pseudogenized in the collagen-like region (red lines in CLR region) (Fig. 4D through F) and were distributed among classical clades C1–C5 and cryptic clade C-I. The low abundance of these alleles suggests that their mutations are not subjected to selective pressure to thrive (Tables S10 to S12).

### *bclA* allele variability

**Fig 5:**
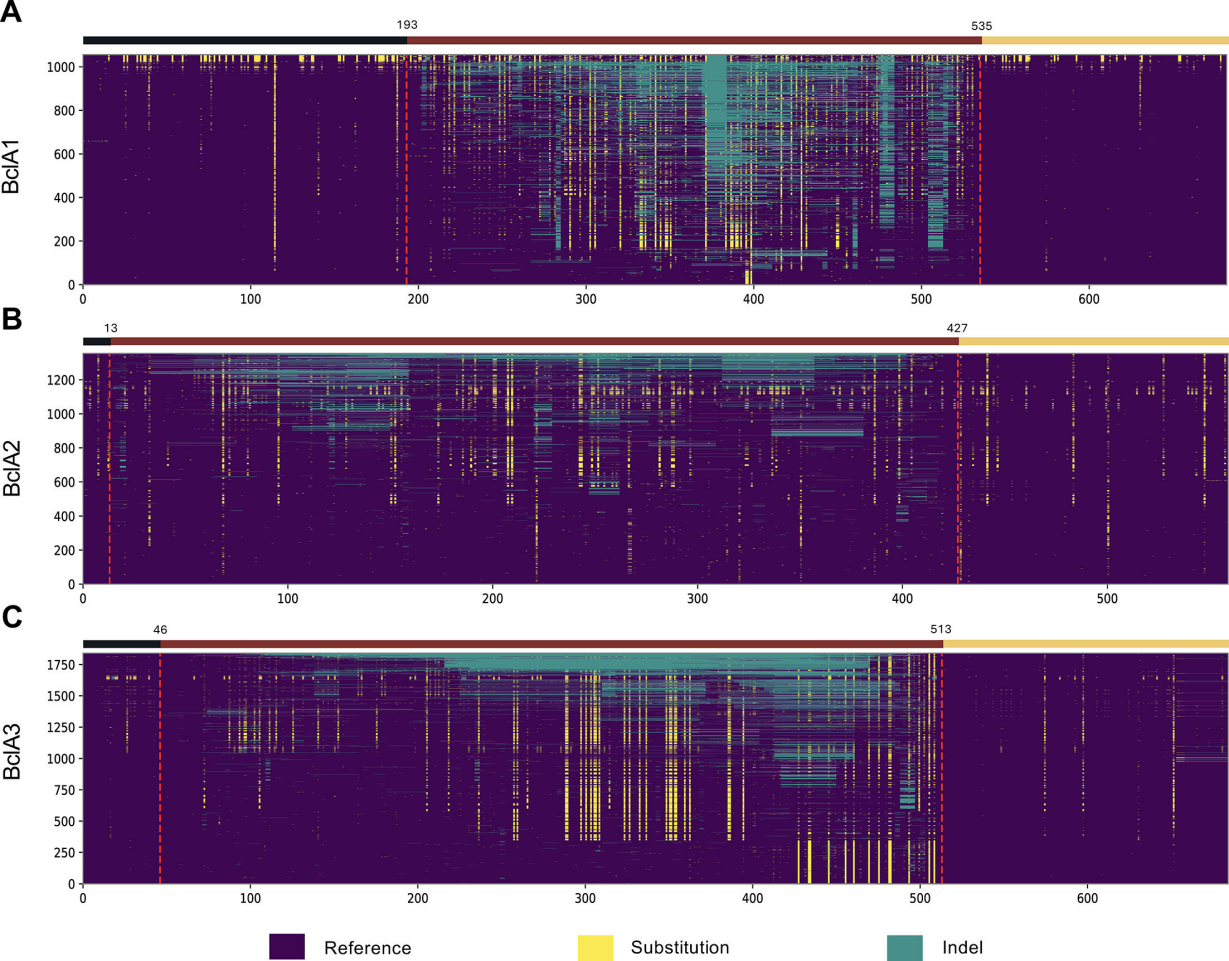
Comparison of *C. difficile* BclA1, BclA2, and BclA3 unique alleles. Unique alleles were aligned using Clustal Omega against the reference. Truncated, interrupted, or pseudogenized sequences were not considered in this analysis. Heatmap displaying positions that differ from or do not differ from the reference sequence are depicted for (**A**) BclA1, Ref. genome: CLO_CA1356AA; (**B**) BclA2, Ref. genome: CLO_AA3432AA; and (**C**) BclA3, Ref. genome: CLO_AA0395AA. Dashed red lines mark the division between BclA domains. NTD, N-terminal domain (black), CLR, collagen-like region (maroon); and CTD, C-terminal domain (yellow).

To further identify differences at the amino acid level of *C. difficile* BclA and define the types, a selection of *bclA* genes was used. To do this, deduplicated complete genes were extracted to obtain a set of unique alleles for *bclA1* (*n* = 1,057, Table S13), *bclA2* (*n* = 1,355, Table S14), and *bclA3* (*n* = 1,842, Table S15). For each *bclA* subset, sequences were aligned against a reference sequence that presented the least differences compared to all corresponding genes extracted: *bclA1* (CLO_CA1356AA) (Fig. 5A), *bclA2* (CLO_AA3432AA) (Fig. 5B), and *bclA3* (CLO_AA0395AA) (Fig. 5C). The alignment was based on codon-aligned amino acids of each BclA set. BclA proteins presented highly conserved NTD and CTD domains with few preserved substitutions distributed throughout (yellow lines). Remarkably, the CLR region showed the most substitutions and indels, making it a highly variable region for BclA collagen-like proteins (Fig. 5). The ANI analysis revealed high variability in the full lengths of BclA1, BclA2, and BclA3 across all clades (Fig. S13A through C); however, both terminal domains showed high conservation within classical clades (Fig. S13D through I). These findings suggest that classical clade-BclA types are very similar, while cryptic BclAs are more divergent.

### Restoration of clade C1 BclA1 into a clade C2 strain reveals subtle effects on growth and sporulation

**Fig 6:**
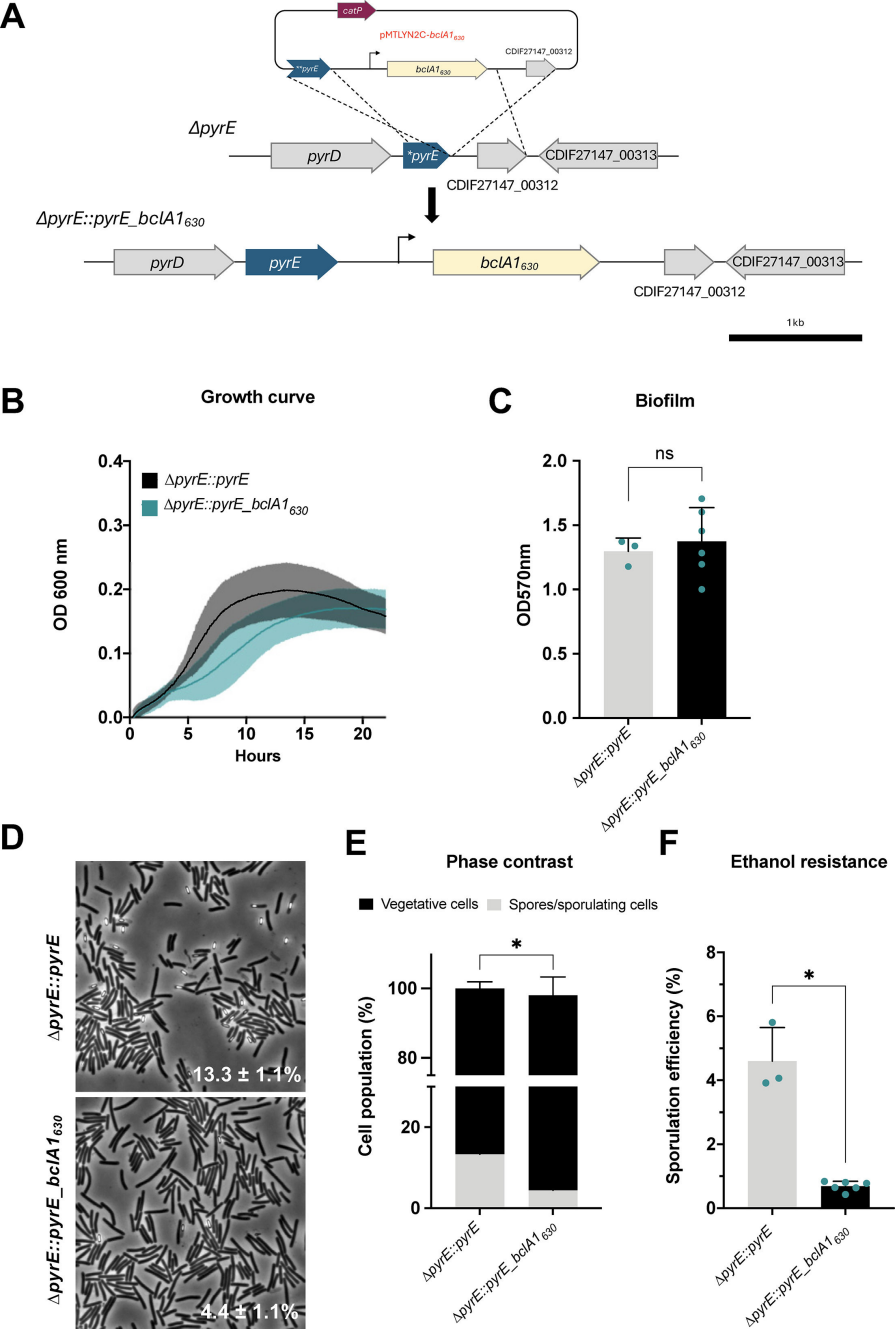
Effect of complementation of *bclA1_630_* in R20291 strain in growth, biofilm formation, and sporulation. (**A**) Schematic representation of *bclA1_630_* integration under the *pyrE* locus. (**B**) Growth curve of *C. difficile ∆pyrE::pyrE* (black) and *∆pyrE::pyrE_bclA1_630_* (teal) isolates in BHIS media over 22 h. (**C**) Biofilm formation of *C. difficile ∆pyrE::pyrE* (gray) and *∆pyrE::pyrE_bclA1_630_* (black) was evaluated at 24 h. Biofilm was stained with 0.1% crystal violet and measured at OD 570 nm. (**D**) Sporulation efficiency evaluated by phase contrast microscopy images of 24 h sporulating cultures grown in 70:30 sporulation media agar of *C. difficile ∆pyrE::pyrE* and *∆pyrE::pyrE_bclA1_630_*. Numbers on the lower right corner correspond to the percentage of spores/sporulating cells in the sample at that time; at least 500 spores/cells were counted from each sample. (**E**) Graphic representation of sporulation efficiency as measured by phase contrast. Percentage of vegetative cells (black) versus spores/sporulating cells (gray) was plotted. (**F**) Sporulation efficiency evaluated by ethanol-resistant sporulation assay of 24 h sporulating cultures grown in 70:30 sporulation media agar of *C. difficile ∆pyrE::pyrE* and *∆pyrE::pyrE_bclA1_630_*. Represented values in panels B, C, D, E, and F are the averages of at least three biological replicates. The means and standard errors are shown. An unpaired *t*-test was used for panels C, E, and F. *P < 0.05. ns, not significant.

Since pseudogenization of *bclA1* (BclA1_48_) is highly prevalent in the genomes of clades C2 and C3 (Fig. 4A, D, and G; Table S8 and S10), the effect that a truncated BclA1 has on growth, sporulation, and spore ultrastructure of *C. difficile* was explored. First, a full-length *bclA1* gene from strain 630 with its native promoter (clade C1) was introduced into the *pyrE* locus of strain R20291 (clade C2) through allelic exchange (Fig. 6A; Fig. S14). The complemented strain showed a slight delay in growth in BHIS broth compared to the wild-type strain (Fig. 6B) but no significant difference in biofilm formation (Fig. 6C). However, a significant decrease (*P* < 0.05) in sporulation was evidenced in the complemented strain (4.4% ± 1.1%) when compared to wild-type (13.3% ± 1.1%) in a 24 h sporulating culture analyzed under phase contrast microscopy (Fig. 6D and E), as well as by ethanol resistance (3.9% ± 0.4%, *P* < 0.05) (Fig. 6F). These results indicate that the introduction of BclA1 in a *bclA1*-lacking clade C2 strain has a slight impact on physiology and sporulation of *C. difficile*.

### Clade C1 BclA1 decreases hair length upon restoration into clade C2 spores

**Fig 7:**
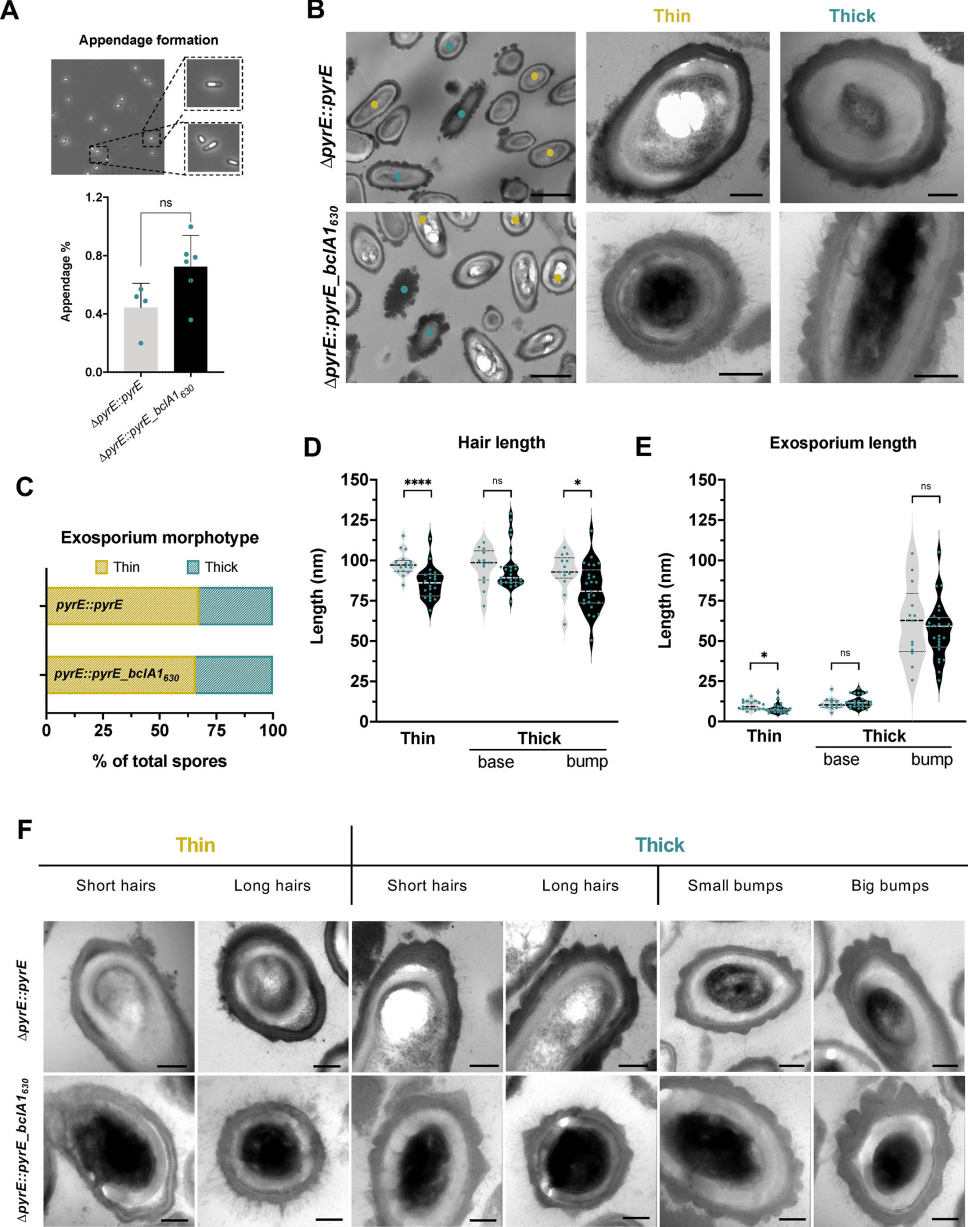
Ultrastructure of R20291-*bclA1_630_*-complemented spores. (**A**) Appendage formation in *C. difficile ∆pyrE::pyrE* and *∆pyrE::pyrE_bclA1_630_* spores. A representative phase contrast microscopy image of two spores with appendages is shown. Graphic representation of appendage formation, at least 400 spores were counted from each strain. The means and standard errors for at least three biological replicates are shown. (**B**) Transmission electron microscopy analysis of *C. difficile ∆pyrE::pyrE* and *∆pyrE::pyrE_bclA1_630_* spores. Yellow and teal circles correspond to thin and thick exosporium spores, respectively. Scale bar: 1 µm. Middle and right panels correspond to representative electron micrographs of thin-exosporium and thick-exosporium morphotypes. Scale bar: 200 nm. (**C**) Thin (yellow) and thick (teal) exosporium morphotypes were quantified in each strain. At least 60 spores were counted from each strain. (**D**) Hair-like projection length and (**E**) electron-dense layer thickness in both thin and thick exosporium morphotypes of *C. difficile ∆pyrE::pyrE* and *∆pyrE::pyrE_bclA1_630_* spores were measured. Each dot represents an average of three individual measurements per spore. For *∆pyrE::pyrE*, 17 thin and 13 thick spores were analyzed, and for *∆pyrE::pyrE_bclA1_630_*, a total of 21 thin and 25 thick spores were measured. (**F**) Representative electron micrographs of outlier phenotype for hair length and bump thickness are shown for both *∆pyrE::pyrE* and *∆pyrE::pyrE_bclA1_630_* thin and thick spores. An unpaired *t*-test was used for panel A, and an unpaired nonparametric Mann-Whitney *U*-test was used for panels D and E. *P < 0.05; **P < 0.01; ***P < 0.001; and ****P < 0.0001. ns, not significant.

Phase contrast microscopy analyses revealed no differences in appendage formation between wild-type (mean = 0.45%) and complemented strain (mean = 0.72%) (Fig. 7A). TEM micrographs of complemented strain showed that spore layers were correctly assembled and presented hair-like projections on the outermost exosporium layer (Fig. 7B). Similarly, the exosporium morphotype distribution reflected expected wild-type levels for thin (~70%) and thick (~30%) (61–63) (Fig. 7C). However, complementation of BclA1 led to a significant decrease in hair length in thin spores and in the electron-dense bumps of thick spores of ~11.8 and ~9.9 nm, respectively. Additionally, although no major difference was seen in the exosporium length when comparing thick wild-type and complemented spores, a slight but significant decrease in the thickness of the electron-dense layer was observed upon comparing thin wild-type and complemented spores, where the *bclA1_630_* complementation reduced the thickness of the exosporium by ~1.8 nm (Fig. 7E), although this slight decrease seems biologically irrelevant. A noteworthy observation was a wide range of lengths for both the hairs, in thin and thick spores, and the bumps in thick spores for both wild-type and complemented strain, reflecting the extensive ultrastructural variability of *C. difficile* spores (Fig. 7F). Altogether, these results indicate that complementation of full-length BclA1 into the R20291 strain decreases the length of the hairs of *C. difficile* spores and the extensive variability of the exosporium and hairs.

## DISCUSSION

*C. difficile* spores possess an outermost exosporium layer that serves as the initial point of contact with the host. Recent research has revealed the ultrastructural diversity, molecular composition, and functional characteristics of this outer layer (11, 13–17, 66, 74, 75). Extensive studies have shown that BclA proteins, distinguished by their collagen-like domains, are key contributors to the assembly and structural organization of the exosporium layer in spores of the *Bacillus cereus* group (18, 21, 76–80). In contrast, the specific roles and functional implications of BclA-like proteins in *C. difficile* spores remain less clearly defined (14, 16, 30, 66). Partial characterization in at least two *C. difficile* strains has indicated that these proteins contribute to colonization, interactions with host tissues, spore persistence, and recurrence of the disease (14, 16, 30).

In this study, we expanded our insights into BclA in *C. difficile* by comprehensively analyzing the distribution and variability of the *bclA* ortholog family across more than 25,000 *C. difficile* genomes from diverse phylogenetic clades, identifying key conserved and divergent regions. Furthermore, AF3 modeling was employed to structurally characterize all three predicted BclA proteins. This work also addressed frequent pseudogenization events affecting the *bclA* genes, particularly *bclA1*, and experimentally evaluated the functional consequences of reintroducing a full-length *bclA1* into a strain with a pseudogenized variant. Results of this work also offer several major conclusions that collectively advance the understanding of the evolutionary dynamics and functional significance of BclA proteins in *C. difficile* spore biology and pathogenesis.

A first contribution of this work is that structural modeling reveals that all three *C. difficile* BclA have a globular CTD capable of homotrimerizing. Indeed, AF3 results provide several insights with biological relevance useful for future hypothesis-guided studies. The fact that AF3 was able to predict, to some extent, the NTD of BclA1, but not BclA2 or BclA3, as a globular domain could be attributed to the extensive length and/or physicochemical properties of the NTD of BclA1 compared to the shorter BclA2 and BclA3 NTDs. All three NTDs were previously shown to guide tdTomato to the surface of *C. difficile* spores (66), yet the distinctive AF3 predictions suggest that the mechanisms of attachment might differ substantially. Alternatively, similarly to *B. anthracis*, it is plausible that the large NTD of BclA1 undergoes extensive post-translational processing during exosporium assembly and prior to attachment to the basal layer (81). While AF3 failed to resolve with confidence the CLR region for all three BclAs, the CTD was strongly predicted to fold as a globular domain, and more importantly, it is capable of forming homotrimers with high confidence. These predictions align with experimental evidence that collagen-like proteins tend to trimerize and that this is guided by the terminal domains (67). However, unlike mammalian collagen proteins that can form heterotrimers, the heterotrimer predictions of different configurations of BclA yielded low to null molecular interactions, suggesting that heterotrimerization between BclA homologs does not occur in *C. difficile*. By contrast, these results support the hypothesis that during the assembly of the exosporium, the hair-like projections are formed as homotrimers of BclA, which are attached to the base via their NTD (66). Indeed, at least in strain R20291, BclA3 is critical for the formation of these hairs (16), although the role of BclA2 in hair formation remains unknown. Despite the usefulness of these results, there are several limitations in AF3 prediction that impact the inferences of this work (44). For example, while AF3 is able to resolve globular domains, which is consistent with high confidence predictions of BclAs CTDs, it provides low confidence for intrinsically disordered sequences such as the collagen-like regions of all three BclAs. In addition, AF3 can predict fake structural orders in disordered regions (44), which could be the case of the prediction of NTD of BclA1. Moreover, the single static structure predicted by AF3 does not mimic its dynamic behavior in solution (44), which might impact how the predicted models in this work behave in aqueous environments such as those found *in vivo*. Despite these limitations, these AF3 predictions serve for further hypothesis-guided studies to understand how homotrimerization occurs and dissect the functional role of homotrimeric BclAs versus monomeric BclA in *C. difficile* spore biology.

A major finding of this study is the extensive variability in the presence, sequence conservation, and integrity of *bclA* orthologs across *C. difficile* clades C1 through C5 and the cryptic clades. While *bclA2* and *bclA3* were broadly conserved across most clades, *bclA1* exhibited substantial variability, including a highly prevalent pseudogenization in multiple lineages, particularly within clades C2 and C3. Clade C5 is considered an early ancestor of classical clades (40), lacking *bclA1* and *bclA3*, while all three *bclA* orthologs were absent in clade C3 genomes. These patterns suggest that selective pressures have driven divergent evolutionary trajectories for spore surface proteins, possibly linked to clade-specific ecological niches or host interactions. It is also tempting to hypothesize that the *bclA* patterns observed across clades might be tied to morphological differences in the hair-like projections. Prior work in strain R20291 demonstrates that BclA3 is essential for hair-like formation, yet it is unclear whether BclA2 contributes to the hair structure (16). Based on these findings, a tentative hypothesis is that members of clade C3, lacking all *bclA* alleles, would produce hairless spores.

However, the presence of *bclA* orthologs does not necessarily correlate with hairs; for example, spores of strain 630 of clade C1, despite encoding all three *bclA*, exhibit a hairless phenotype (62). A limitation of these results is that the *bclA* extraction pipeline worked under the hypothesis that *bclA* orthologs are consistently within the same genetic context. Hence, in the likely scenario that some *bclA* locate to a different loci not identified by conserved flanking regions, it would lead to an overrepresentation of “not found” alleles. Regardless of these limitations, these observations suggest that the formation of hair-like projections in *C. difficile* spores is more complex than previously understood and may involve differential expression of *bclA* orthologs across clades, potentially compensating for the absence or pseudogenization of one or more genes.

The high *bclA* variability observed across clades is likely to lead to distinct functions in virulent traits related to spore-host interactions. Functional studies in different clades have shed insight into their roles (14, 16, 30). For example, work in clade C1 strain 630 (11, 17, 30) shows that *bclA1* is required for proper colonization in mice (30). However, disruption of individual *bclA* led to spores lacking the electron-dense exosporium and a defective spore coat, suggesting a pleiotropic effect on the spore surface (30). By contrast, work on a clade C2 strain (R20291) shows that a single deletion of *bclA3* is sufficient to impair the formation of the hair-like projection without impacting the formation of the lower electron-dense layers and spore coat (16). BclA3 was also associated with persistence and disease recurrence (16). This limited knowledge on *C. difficile* BclA suggests that, depending on the clade, BclA might have distinct niche-specific advantages, underscoring the need to investigate the impact of BclA in different clades.

Another major conclusion this work offers is the striking pattern of sequence conservation and variability across the BclA proteins. In each case, both the NTD and CTD were highly conserved across the more than 25,000 *bclA* alleles analyzed, whereas the central collagen-like regions exhibited substantial sequence diversity. The fact that conserved terminal domains flank a highly variable central region suggests that the preservation of these domains, required for BclA anchoring and structural stability (the NTD and CTD, respectively), allows for evolutionary flexibility in the central collagen-like region. This pronounced variability may be attributed to the repetitive GXY triplet motif that results in underlying DNA sequence repeats. Such repeats are known to increase genetic instability through replication slippage or unequal recombination, which can result in insertions, deletions, or sequence rearrangements (82). While this was not investigated further, it is likely that in *C. difficile,* the collagen-like domain may be subjected to diversification by host-derived selective pressures, including evasion of host immune responses or adaptation to distinct host environments as observed in *B. anthracis* spores (83, 84). Together, these findings indicate that the collagen-like region is a shared hotspot for adaptive variation across the BclA protein family in *C. difficile*. Additionally, these observations deserve further work to address how CLR variations affect spore adherence, environmental persistence, or host colonization dynamics.

A notable finding from our analysis is the pattern of *bclA1* pseudogenization across *C. difficile* clades. While pseudogenization events in *bclA* genes were generally rare, occurring at a frequency below 1% across most clades, a striking exception was observed in clades C2 and C3. In these clades, most strains carried a conserved pseudogenized *bclA1* allele that encodes only a short 48-amino acid peptide corresponding to the NTD of BclA1. Despite the truncation, this NTD fragment appears to retain the structural features necessary for anchoring to the spore surface (30). The conservation of this pseudogenized form is particularly intriguing, as it suggests that full-length BclA1 may be dispensable in these lineages, or that the truncated form fulfills a distinct, potentially clade-specific function. This notion might also be true for the pseudogenized *bclA2* and *bclA3* alleles in clade C3 strains. This raises questions about the evolutionary pressures shaping *bclA* function and whether the presence of only the anchoring domain represents an adaptive modification.

The reintroduction of a full-length *bclA1* gene into a clade C2 strain with a conserved pseudogenized allele, followed by functional characterization, offers insights into the role of this protein in *C. difficile* spore ultrastructure and biology. While no significant changes were observed in biofilm formation, a slight decrease in growth and sporulation was evidenced. A potential explanation for the delayed growth could be due to leaky transcription from the *pyrE* promoter toward the downstream inserted *bclA1* gene, leading to the expression of an energetically expensive protein during growth and sporulation, although this is very unlikely. Regarding spore architecture, restoration of *bclA1* influenced the spore surface structure, albeit had no impact on the ratio of thick- to thin-exosporium spores, indicating that the mere expression of BclA1 does not affect the regulation of these two distinctive exosporium expression/assembly pathways (17). However, the fact that restoration of full-length *bclA1* shortened the length of the hairs by ~10%–12% could indicate that there is a competition for the anchoring site between BclA1 and BclA2 and/or BclA3, resulting in a shortening of the hair-like projections. Although the presence of BclA1 by immunoblotting was not confirmed due to a lack of a proper antibody, further studies addressing the titration of the BclAs on the spore surface would consolidate this notion. BclA1 seems to play a role in exosporium’s structural features, raising the hypothesis that clade-specific *bclA* pseudogenization shapes hair structure and impacts spore virulence. The potential implications of such changes may involve how spores of clade C2 interact with host tissues, environmental surfaces, or immune components, underscoring the nuanced contributions of BclA1 to *C. difficile* biology.

In summary, this work provides the first overview of the diversity of spore surface proteins across *C. difficile* clades. This study also highlights the diversity of the collagen-like BclA proteins in *C. difficile* and demonstrates that they exhibit modular domains defined by conserved N- and C-terminal domains, flanking a highly variable collagen-like region. Intriguingly, alterations in *bclA* alleles in several clinically relevant clades (e.g., clades C2 and C3) likely shape spore surface architecture and lead to clade-specific spore tropism toward host surfaces, ultimately affecting spore virulence traits, topics that are currently being investigated in the lab.

## SUPPLEMENTAL MATERIAL

Supplemental figures aem.01582-25-s0001.pdf Figures S1 to S14.

Supplemental tables aem.01582-25-s0002.xlsx Tables S1 to S15.

ASM does not own the copyrights to Supplemental Material that may be linked to, or accessed through, an article. The authors have granted ASM a non-exclusive, world-wide license to publish the Supplemental Material files. Please contact the corresponding author directly for reuse.
