# Supplementary material for: Phylogenomic analysis of the collagen-like BclA proteins in Clostridioides difficile
Source: Appl Environ Microbiol. 2026 Mar 3;92(5):e01582-25. doi: 10.1128/aem.01582-25 (PMC13188886; doi:10.1128/aem.01582-25)
Supplement: Supplemental figures — Figures S1 to S14. [file aem.01582-25-s0001.pdf]

Figure S1

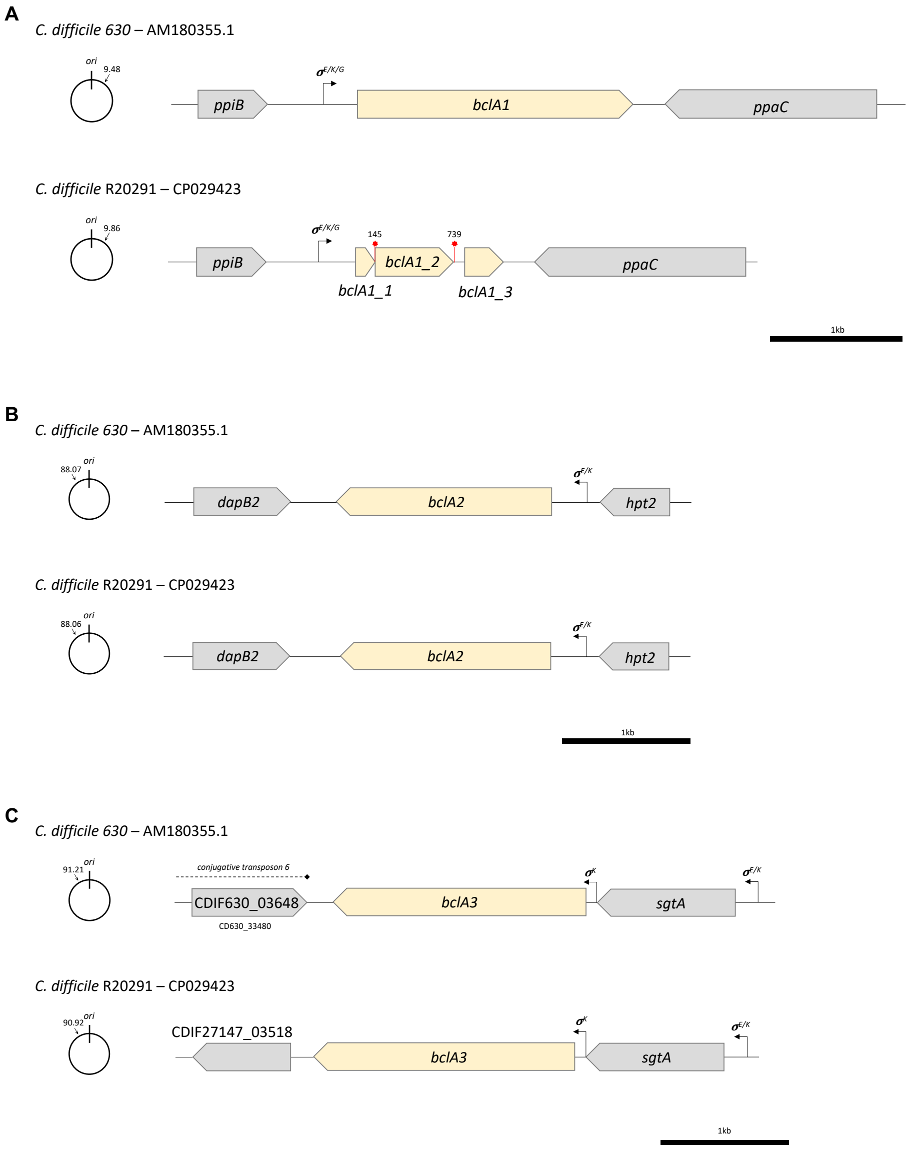

Fig. S1. *C. difficile* 630 and R20291 *bclA* gene schematic representation. (A) *bclA1*$_{630}$ (CD630_03320) is a 2082 bp gene coding a 694 amino acid protein (67.8 kDa). *bclA1*$_{R20291}$ (CDIF27147_00471, CDIF27147_00472, CDIF27147_00473) is represented as a three-segmented ORF (*bclA1_1, bclA1_2 & bclA1_3*) because it is a pseudogenized gene. A nonsense mutation, A145T, generated an early stop codon resulting in a short protein of 48 amino acids (4.7 kDa). Downstream, another nonsense mutation, C739T, also generates an early stop codon and the two extra segments of *bclA1*$_{R20291}$ ORF. *bclA1* possess a σ^K promoter region (Saujet, 2013), and two putative σ^E and σ^G consensus sequences that were found during this work. *bclA1* is flanked upstream by *ppiB* and downstream by *ppaC*. **(B)** *bclA2*$_{630}$ (CD630_32300) is a 1677 bp gene coding a 558 amino acid protein (49.1 kDa). *bclA2*$_{R20291}$ (CDIF27147_03409) is a 1641 bp gene coding a 546 amino acid protein (47.9 kDa). *bclA2* possess a σ^K promoter region (Saujet, 2013) and a putative σ^E consensus sequence that was found during this work. *bclA2* is flanked upstream by *hpt2* and downstream by *dapB2*. **(C)** *bclA3*$_{630}$ (CD630_33490) is a 1986 bp gene coding a 661 amino acid protein (58.3 kDa). *bclA3*$_{R20291}$ (CDIF27147_03519) is a 2037 bp gene coding a 678 amino acid protein (59.9 kDa). *bclA3* is the second gene of an operon with an upstream *sgtA* gene coding for a glycosyl transferase (CD630_33500, CDIF27147_03520). The operon possesses a σ^K promoter region, and an additional σ^K promoter region (Saujet, 2013) immediately upstream *bclA3* and a putative σ^E consensus sequence that was found upstream the operon during this work. *bclA3* is flanked upstream by *sgtA* and downstream by CDIF27147_03518. The genomes of *C. difficile* 630 (AM180355.1) and *C. difficile* R20291 (CP029423) were used as references. Scale bar: 1 kb.

Figure S2

```
                                         NTD
BclA1_630        1 MRNIILYLNDDTFISKKYPDKNFSNLDYCLIGSKCSNSFVKEKLITFFKV     50
                   ||.|||||||||||||||||||||||||||||||||||||||||||||||
BclA1_R20291     1 MRKIILYLNDDTFISKKYPDKNFSNLDYCLIGSKCSNSFVKEKLITFFKV     50

BclA1_630       51 RIPDILKDKSILKAELFIHIDSNKNHIFKEKVDIEIKRISEYYNLRTITW    100
                   ||||||||||||||||||||||||||||||||||||||||||||||||||
BclA1_R20291    51 RIPDILKDKSILKAELFIHIDSNKNHIFKEKVDIEIKRISEYYNLRTITW    100

BclA1_630      101 NDRVSMENIRGYLPIGISDTSNYICLNITGTIKAWAMNKYPNYGLALSLN    150
                   ||||||||||||||||||||||||||||||||||||||||||||||||||
BclA1_R20291   101 NDRVSMENIRGYLPIGISDTSNYICLNITGTIKAWAMNKYPNYGLALSLN    150

BclA1_630      151 YPYQILEFTSSRGCNKPYILVTFEDRIIDNCYPKCECPPIRITGPMGPRG    200
                   |||||.||||||.||||||||||||||||||||||||.||||||||||||
BclA1_R20291   151 YPYQIFEFTSSRDCNKPYILVTFEDRIIDNCYPKCECLPIRITGPMGPRG    200

                                        CLR
BclA1_630      201 ATGSTGPMGVTGPTGSTGATGSIGPTGPTGNTGATGSIGPTGVTGPTGST    250
                   ||||.||||.
BclA1_R20291   201 ATGSIGPMGA----------------------------------------    210

BclA1_630      251 GATGSIGPTGVTGPTGNTGVTGSIGPTGATGPTGNTGVTGSIGPTGVTGP    300

BclA1_R20291   210 --------------------------------------------------    210

BclA1_630      301 TGNTGEIGPTGATGPTGVTGSIGPTGATGPTGEIGPTGATGATGSIGPTG    350

BclA1_R20291   210 --------------------------------------------------    210

BclA1_630      351 ATGPTGATGVTGEIGPTGEIGPTGATGPTGVTGSIGPTGATGPTGATGEI    400

BclA1_R20291   210 --------------------------------------------------    210

BclA1_630      401 GPTGATGPTGVTGSIGPTGATGPTGATGEIGPTGATGPTGVTGEIGPTGA    450

BclA1_R20291   210 --------------------------------------------------    210

BclA1_630      451 TGPTGNTGVTGEIGPTGATGPTGNTGVTGEIGPTGATGPTGVTGEIGPTG    500

BclA1_R20291   210 --------------------------------------------------    210

BclA1_630      501 NTGATGSIGPTGVTGPTGATGSIGPTGATGATGVTGPTGPTGATGNSSQP    550
                                                        |||||||||||||
BclA1_R20291   211 -------------------------------------TGPTGATGNSSQP    223

                                        CTD
BclA1_630      551 VANFLVNAPSPQTLNNGDAITGWQTIIGNSSSITVDTNGTFTVQENGVYY    600
                   :||||||||||||||||:|||||:||||||||||||.|||||||||||||
BclA1_R20291   224 IANFLVNAPSPQTLNNGNAITGWKTIIGNSSSITVDANGTFTVQENGVYY    273

BclA1_630      601 ISVSVALQPGSSSINQYSFAILFPILGGKDLAGLTTEPGGGGVLSGYFAG    650
                   ||||||||||||||||||||||||||||||||||||||||||||||||||
BclA1_R20291   274 ISVSVALQPGSSSINQYSFAILFPILGGKDLAGLTTEPGGGGVLSGYFAG    323

BclA1_630      651 FLFGGTTFTINNFSSTTVGIRNGQSAGTAATLTIFRIADTVMT    693
                   |||||||||||||||||||||||||||||||||||||||||||
BclA1_R20291   324 FLFGGTTFTINNFSSTTVGIRNGQSAGTAATLTIFRIADTVMT    366
```

Fig. S2. Pairwise sequence alignment (PSA) of *C. difficile* 630 & R20291 BclA1. EMBOSS Needle was used for PSA. A representation of the protein domains is depicted above the sequence. The proteins possess a 51.4% Identity. Repaired BclA1 from R20291 was used for the alignment. N-terminal domain (NTD, Black), Collagen-like region (CLR, Maroon) and C-terminal domain (CTD, Yellow). The genomes of *C. difficile* 630 (AM180355.1) and *C. difficile* R20291 (CP029423) were used as references

Figure S3

```
                                NTD
BclA2_630          1 MSDISGPSLYQDVGPTGPTGATGPTGPTGPRGATGATGANGITGPTGNTG     50
                     ||||||||||||||||||||||||||||||||||||||||||||||||||
BclA2_R20291       1 MSDISGPSLYQDVGPTGPTGATGPTGPTGPRGATGATGANGITGPTGNTG     50

BclA2_630         51 ATGANGITGPTGNMGATGPNGTTGSTGPTGNTGATGANGITGPTGNTGAT    100
                     ||||||||||||||||||.||||||||||||||||||||||||||||.||||
BclA2_R20291      51 ATGANGITGPTGNMGATGANGTTGSTGPTGNTGATGANGITGPTGATGAT    100

BclA2_630        101 GANGITGPTGNKGATGANGITGSTGPTGNTGATGANGITGPTGNTGATGA    150
                     ||||||||||||||||||||   |||||.|||||||||||||||||||||
BclA2_R20291     101 GANGITGPTGNKGATGANGI---TGPTGATGATGANGITGPTGNTGATGA    147

BclA2_630        151 TGPTGLTGATGATGANGITGPTGNTGATGANGVTGATGPTGNTGATGPTG    200
                     .|.|||||||||||||||||||||.|||||||||||||||||||||||||
BclA2_R20291     148 NGATGLTGATGATGANGITGPTGATGATGANGVTGATGPTGNTGATGPTG    197

                                                CLR
BclA2_630        201 SIGATGATGTTGATGPIGATGATGADGEVGPTGAVGATGPDGLVGPTGPT    250
                     |||||||.|.||||||||||         |||||||||||||||||||||
BclA2_R20291     198 SIGATGANGVTGATGPIGAT---------GPTGAVGATGPDGLVGPTGPT    238

BclA2_630        251 GPTGATGANGLVGPTGPTGATGANGLVGPTGATGATGVAGAIGPTGAVGA    300
                     ||||||||||||||||||||||||||||||||||||||||||||||||||
BclA2_R20291     239 GPTGATGANGLVGPTGPTGATGANGLVGPTGATGATGVAGAIGPTGAVGA    288

BclA2_630        301 TGPTGADGAVGPTGATGATGANGATGPTGAVGATGANGVAGPIGPTGPTG    350
                     ||||||||||||||||||||||||||||||||||||||||||||||||||
BclA2_R20291     289 TGPTGADGAVGPTGATGATGANGATGPTGAVGATGANGVAGPIGPTGPTG    338

BclA2_630        351 ENGVAGATGATGATGANGATGPTGAVGATGANGVAGAIGPTGPTGANGAT    400
                     .||||||||||||||||||||||||||||||||||||.|||||||||||.|
BclA2_R20291     339 ANGVAGATGATGATGANGATGPTGAVGATGANGVAGPIGPTGPTGANGTT    388

                                                         CTD
BclA2_630        401 GATGATGATGANGATGPTGATGATGVLAANNAQFTVSSSSLVNNTLVTFN    450
                     |||||||||||||||||||||||||||||||||||||||.||||||||
BclA2_R20291     389 GATGATGATGANGATGPTGATGATGVLAANNAQFTVSSSSLGNNTLVTFN    438

BclA2_630        451 SSFINGTNITFPTSSTINLAVGGIYNVSFGIRATLSLAGFMSITTNFNGV    500
                     ||||||||||||||||||||||||||||||||||.|||||||||||||||
BclA2_R20291     439 SSFINGTNITFPTSSTINLAVGGIYNVSFGIRAILSLAGFMSITTNFNGV    488

BclA2_630        501 TQNNFIAKAVNTLTSSDVSVSLSFLVDARAAAVTLSFTFGSGTTGTSAAG    550
                     .||||||||||||||||||||||||||||||||||||||||||||||.||
BclA2_R20291     489 AQNNFIAKAVNTLTSSDVSVSLSFLVDARAAAVTLSFTFGSGTTGTSPAG    538

BclA2_630        551 YVSVYRIQ     558
                     ||||||||
BclA2_R20291     539 YVSVYRIQ     546
```

Fig. S3. Pairwise sequence alignment (PSA) of *C. difficile* 630 & R20291 BclA2. EMBOSS Needle was used for PSA. A representation of the protein domains is depicted above the sequence. The proteins possess a 95.2% Identity. N-terminal domain (NTD, Black), Collagen-like region (CLR, Maroon) and C-terminal domain (CTD, Yellow). The genomes of *C. difficile* 630 (AM180355.1) and *C. difficile* R20291 (CP029423) were used as references.

Figure S4

```
                                NTD
BclA3_630          1 MSRNKYFGPFDDNDYNNGYDKYDDCNNGRDDYNSCDCHHCCPPSCVGPTG     50
                     ||||||||||||||||||||||||||||||||||||||||||||||||||
BclA3_R20291       1 MSRNKYFGPFDDNDYNNGYDKYDDCNNGRDDYNSCDCHHCCPPSCVGPTG     50

BclA3_630         51 PMGPRGRTGPTGPTGPTGPGVGATGPTGPTGPTGPTGNTGNTGATGLRGP    100
                     ||||||||||||||||||||||.|||||||||||||||||||||||||||
BclA3_R20291      51 PMGPRGRTGPTGPTGPTGPGVGGTGPTGPTGPTGPTGNTGNTGATGLRGP    100

BclA3_630        101 TGATGATGPTGATGAIGFGV---TGPTGPTGATGATGADGVTGPTGPTGA    147
                     |||||.||||||||||||||   |||||||||||||||||||||||||||
BclA3_R20291     101 TGATGGTGPTGATGAIGFGVTGPTGPTGPTGATGATGADGVTGPTGPTGA    150

BclA3_630        148 TGADGITGPTGATGATGFGVTGPTGPTGATGVGVTGATGLIGPTGATGTP    197
                     ||||||||||||||||||||||||||||||||||||||||||||||||||
BclA3_R20291     151 TGADGITGPTGATGATGFGVTGPTGPTGATGVGVTGATGLIGPTGATGTP    200

                                  CLR
BclA3_630        198 GATGPTGAIGATGIGITGPTGATGATGADGATGVTGPTGPTGATGADGVT    247
                     ||||||||||||||||||||||||||||||||||||||||||||||||||
BclA3_R20291     201 GATGPTGAIGATGIGITGPTGATGATGADGATGVTGPTGPTGATGADGVT    250

BclA3_630        248 GPTGATGATGIGITGPTGPTGATGIGITGATGLIGPTGATGTPGATGPTG    297
                     ||||||||||||||||||.||||||||||||||||||||||..|||||||
BclA3_R20291     251 GPTGATGATGIGITGPTGATGATGIGITGATGLIGPTGATGATGATGPTG    300

BclA3_630        298 ATGPTG-VGVTGATGATGATGADGATGVTGPTGATGATGANGLVGPTGAT    346
                     .||.|| .|:.|.|||||.||||||||.||.|||||.|||:|||||||||
BclA3_R20291     301 VTGATGAAGLIGPTGATGVTGADGATGATGATGATGPTGADGLVGPTGAT    350

BclA3_630        347 GAAGTPGATGPTGATGPTGVGITGATGATGATGPTGADGATGPTGATGNT    396
                     ||.|..|..||||.||.||||||||||||||||||||||..|||||||.|
BclA3_R20291     351 GATGADGLVGPTGPTGATGVGITGATGATGATGPTGADGLVGPTGATGAT    400

BclA3_630        397 GADGVAGP---TGATGNTGADGATGPTGATGATGADGATGPTGATGATGV    443
                     ||||||||   ||||||||||||||||||||.|||||..||||||||||:
BclA3_R20291     401 GADGVAGPTGATGATGNTGADGATGPTGATGPTGADGLVGPTGATGATGL    450

BclA3_630        444 AGATGATGP---TGATGADGATGPTGATGATGADGATGPTGATGATGVTG    490
                     |||||||||   ||.||||||||.|||||.|||||..||||||||   ||
BclA3_R20291     451 AGATGATGPIGATGPTGADGATGATGATGPTGADGLVGPTGATGA---TG    497

BclA3_630        491 ATGPTGPTGATGATGATGASAIIPFASGIPLSLTTIAGGLVGTPGFVGFG    540
                     |||||||         ||||||||||||||||||||||||||||||||||
BclA3_R20291     498 ATGPTGP---------TGASAIIPFASGIPLSLTTIAGGLVGTPGFVGFG    538

                                  CTD
BclA3_630        541 SSAPGLSIVGGVIDLTNAAGTLTNFAFSMPRDGTITSISAYFSTTAALSL    590
                     ||||||||||||||||||||||||||||||||||||||||||||||||||
BclA3_R20291     539 SSAPGLSIVGGVIDLTNAAGTLTNFAFSMPRDGTITSISAYFSTTAALSL    588

BclA3_630        591 VGSTITITATLYQSTAPNNSFTAVPGATVTLAPPLTGILSVGSISSGIVT    640
                     ||||||||||||||||||||||||||||||||||||||||||||||||||
BclA3_R20291     589 VGSTITITATLYQSTAPNNSFTAVPGATVTLAPPLTGILSVGSISSGIVT    638

BclA3_630        641 GLNIAATAQTPDRQYAI--------------------------    657
                     ||||||||:|   ::.:
BclA3_R20291     639 GLNIAATAET---RFLLVFTATASGLSLVNTVAGYASAGIAIN    678
```

Fig. S4. Pairwise sequence alignment (PSA) of *C. difficile* 630 & R20291 BclA3. EMBOSS Needle was used for PSA. A representation of the protein domains is depicted above the sequence. The proteins possess an 86.9% Identity. N-terminal domain (NTD, Black), Collagen-like region (CLR, Maroon) and C-terminal domain (CTD, Yellow). The genomes of *C. difficile* 630 (AM180355.1) and *C. difficile* R20291 (CP029423) were used as references.

Figure S5

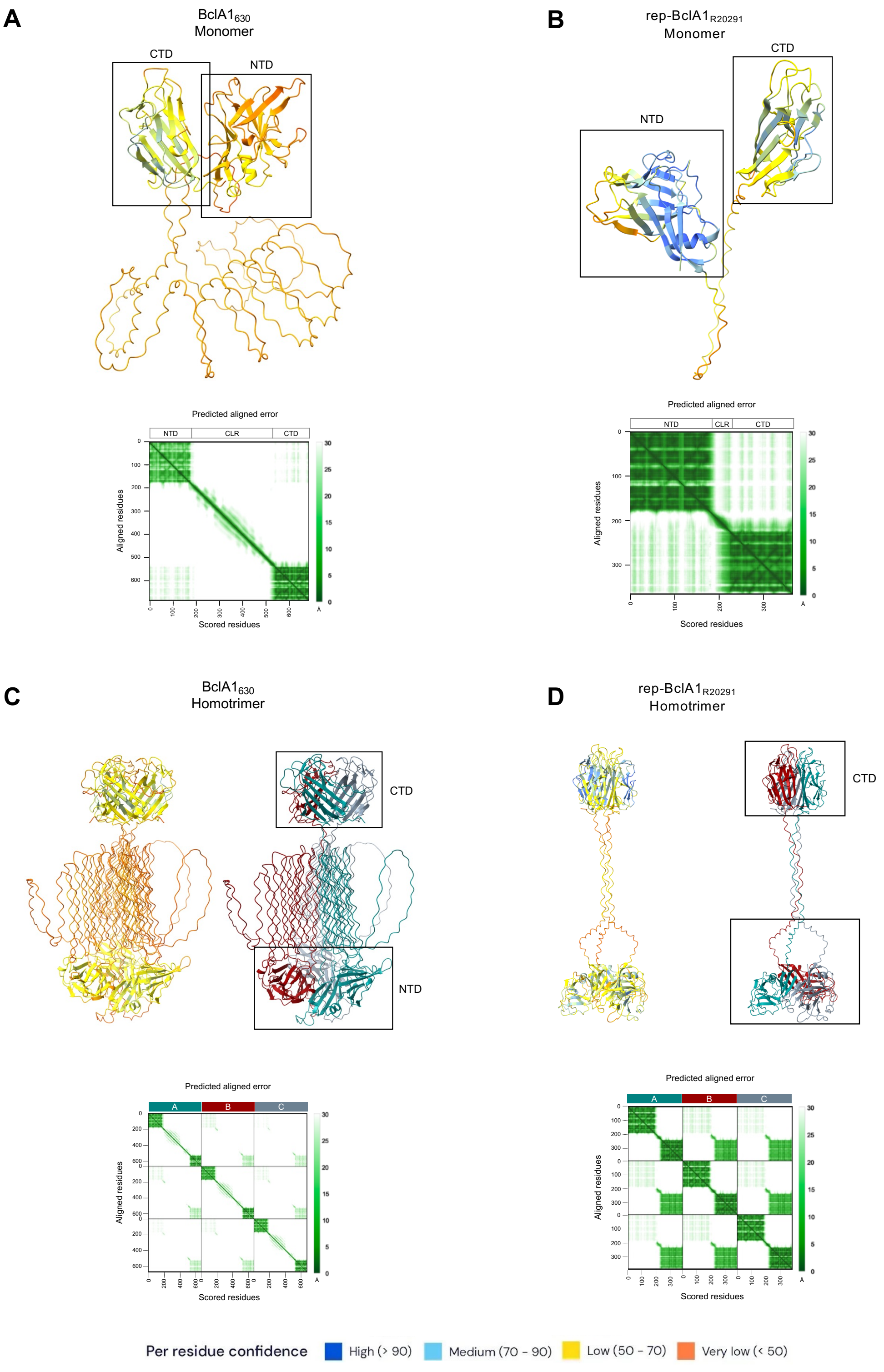

Fig. S5. Full-length BclA1 structure predicted by Alphafold3. Predicted monomeric structure of *C. difficile* **(A)** $BclA1_{630}$, **(B)** rep-$BclA1_{R20291}$, and predicted homotrimer structures of **(C)** $BclA1_{630}$ and **(D)** rep-$BclA1_{R20291}$. Each monomer unit has a different color for easy recognition in trimeric structures. Each model has its respective Predicted aligned error (PAE) plot below. NTD: N-terminal domain, CLR: Collagen-like region, CTD: C-terminal domain.

Figure S6

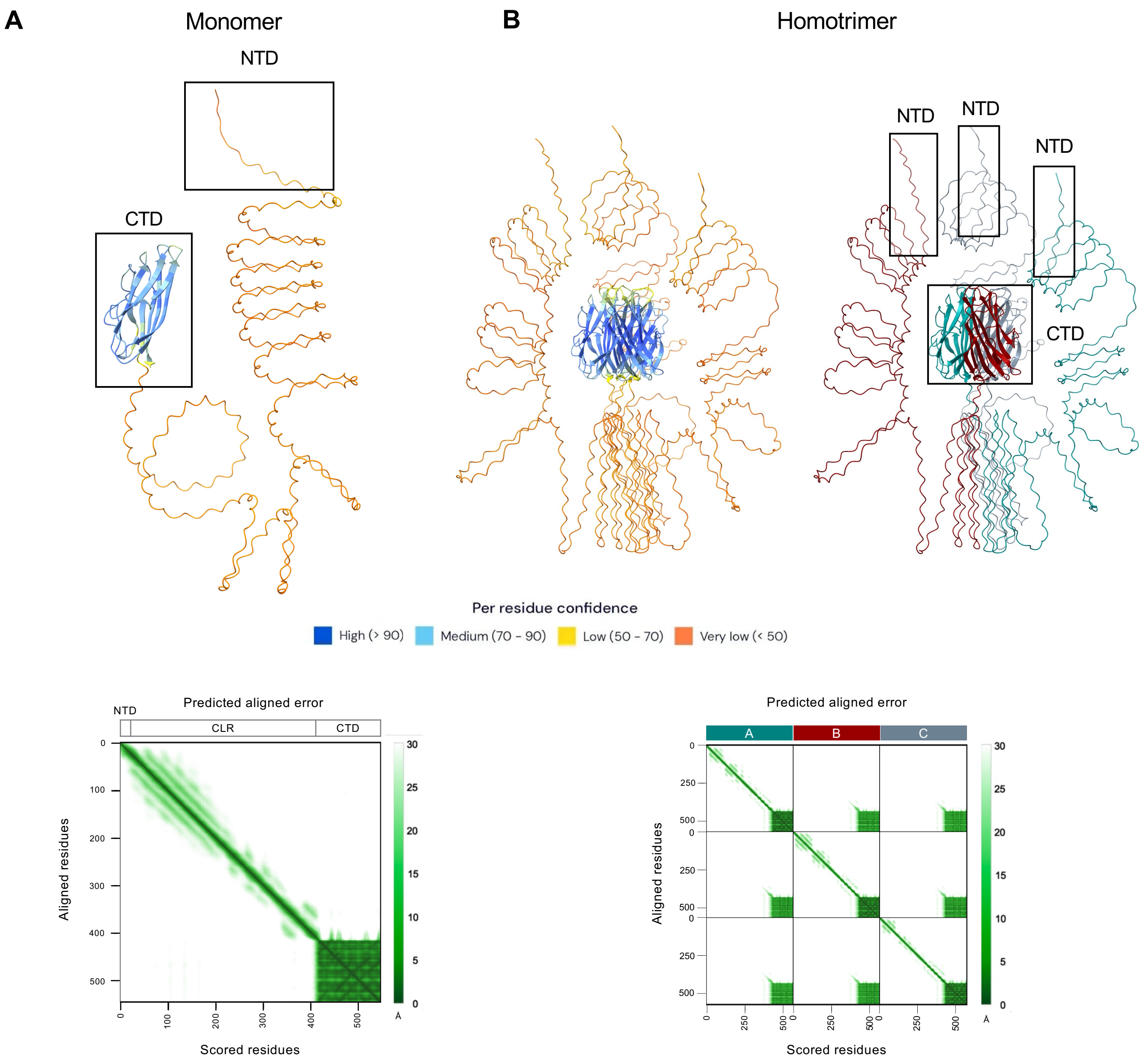

Fig. S6. Full-length BclA2$_{R20291}$ structure predicted by Alphafold3. Predicted structure of *C. difficile* **(A)** BclA2$_{R20291}$ monomer and **(B)** homotrimer complex. Each monomer unit has a different color for easy recognition in trimeric structures. Each model has its respective Predicted aligned error (PAE) plot below. NTD: N-terminal domain, CLR: Collagen-like region, CTD: C-terminal domain.

Figure S7

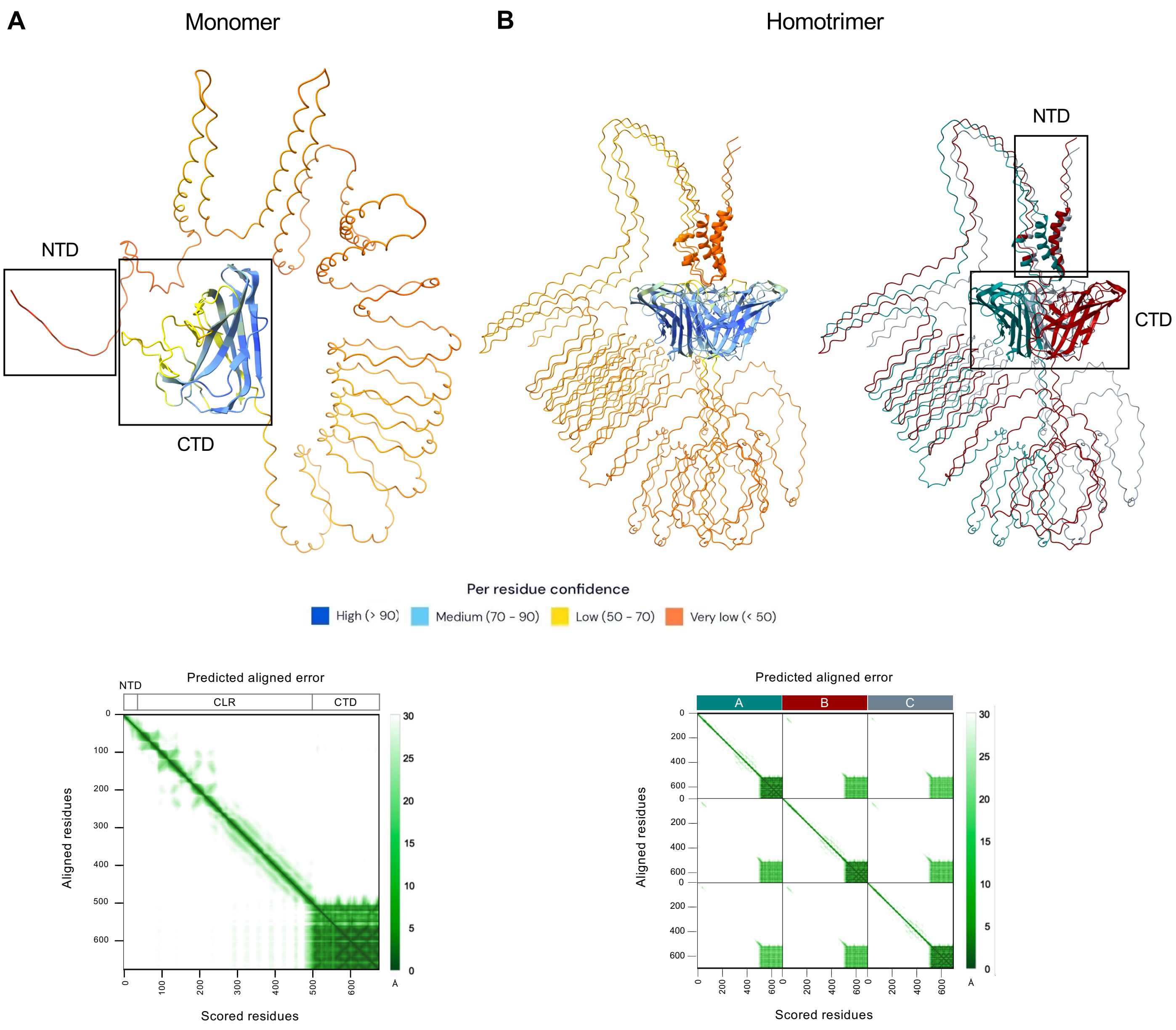

Fig. S7. Full-length BclA3$_{R20291}$ structure predicted by Alphafold3. Predicted structure of *C. difficile* **(A)** BclA3$_{R20291}$ monomer and **(B)** homotrimer complex. Each monomer unit has a different color for easy recognition in trimeric structures. Each model has its respective Predicted aligned error (PAE) plot below. NTD: N-terminal domain, CLR: Collagen-like region, CTD: C-terminal domain.

Figure S8

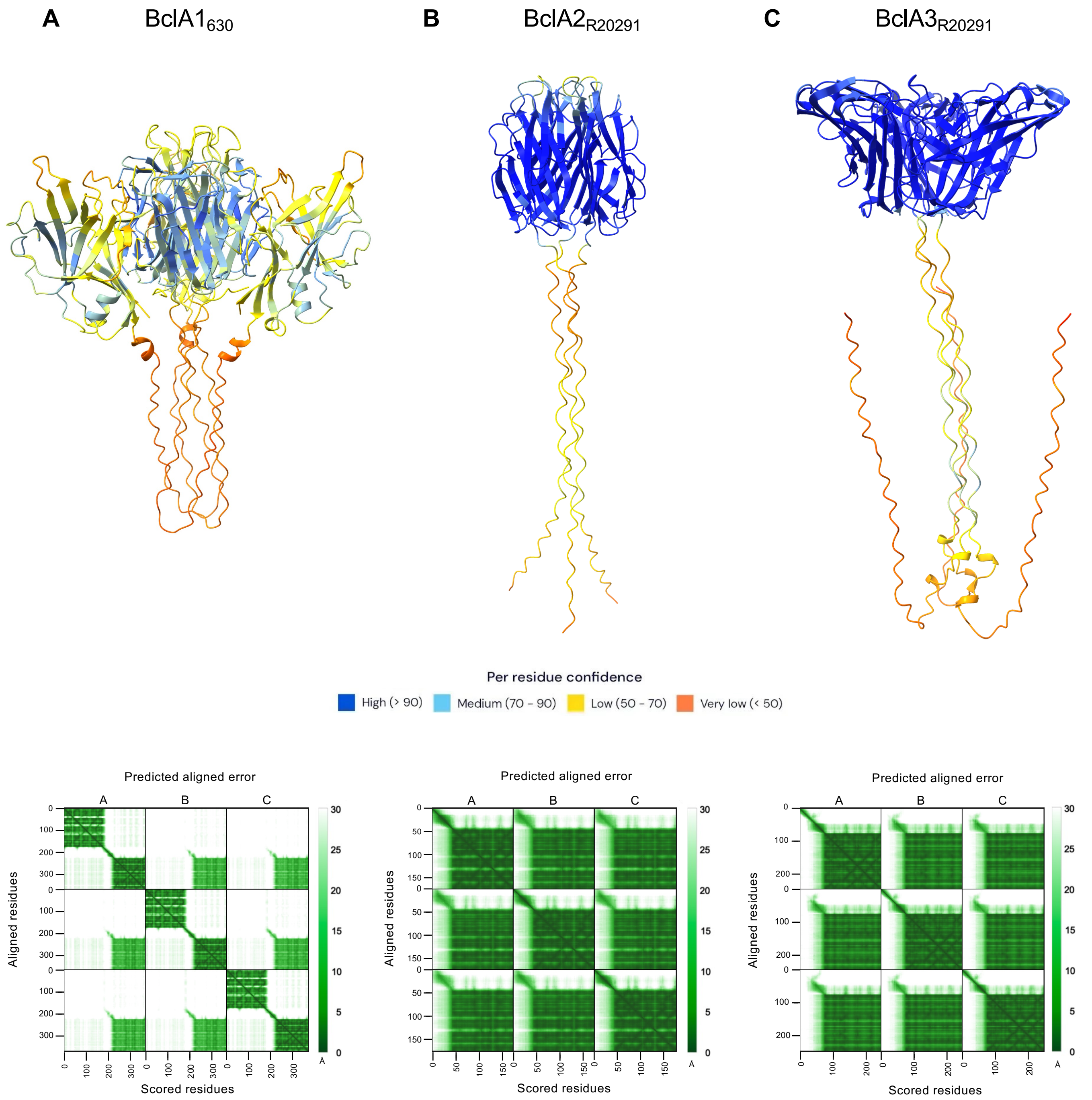

Fig. S8. AlphaFold3 predicted structure of BclA_10GXY homotrimer. Predicted structure of *C. difficile* **(A)** BclA1$_{630}$, **(B)** BclA2$_{R20291}$ and **(C)** BclA3$_{R20291}$ homotrimer complexes. Each model has its respective Predicted aligned error (PAE) plot below.

Figure S9

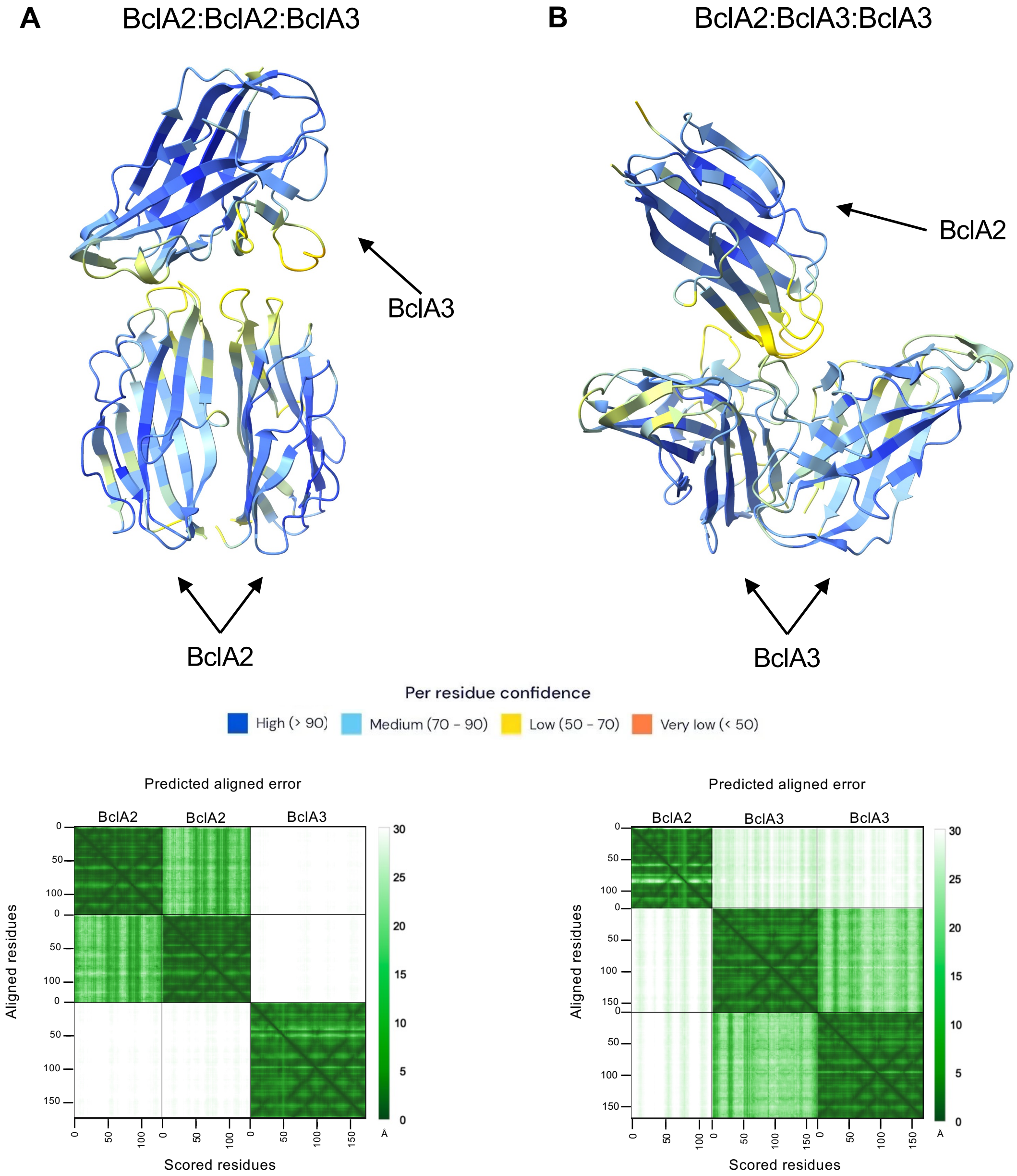

Fig. S9. AlphaFold3 predicted CTD heterotrimer structures from strain R20291. Heterotrimer predicted model composed of **(A)** 2 units of BclA2_CTD and 1 unit of BclA3_CTD or **(B)** 1 unit of BclA2_CTD and 2 unit of BclA3_CTD. Each model has its respective Predicted aligned error (PAE) plot below.

Figure S10

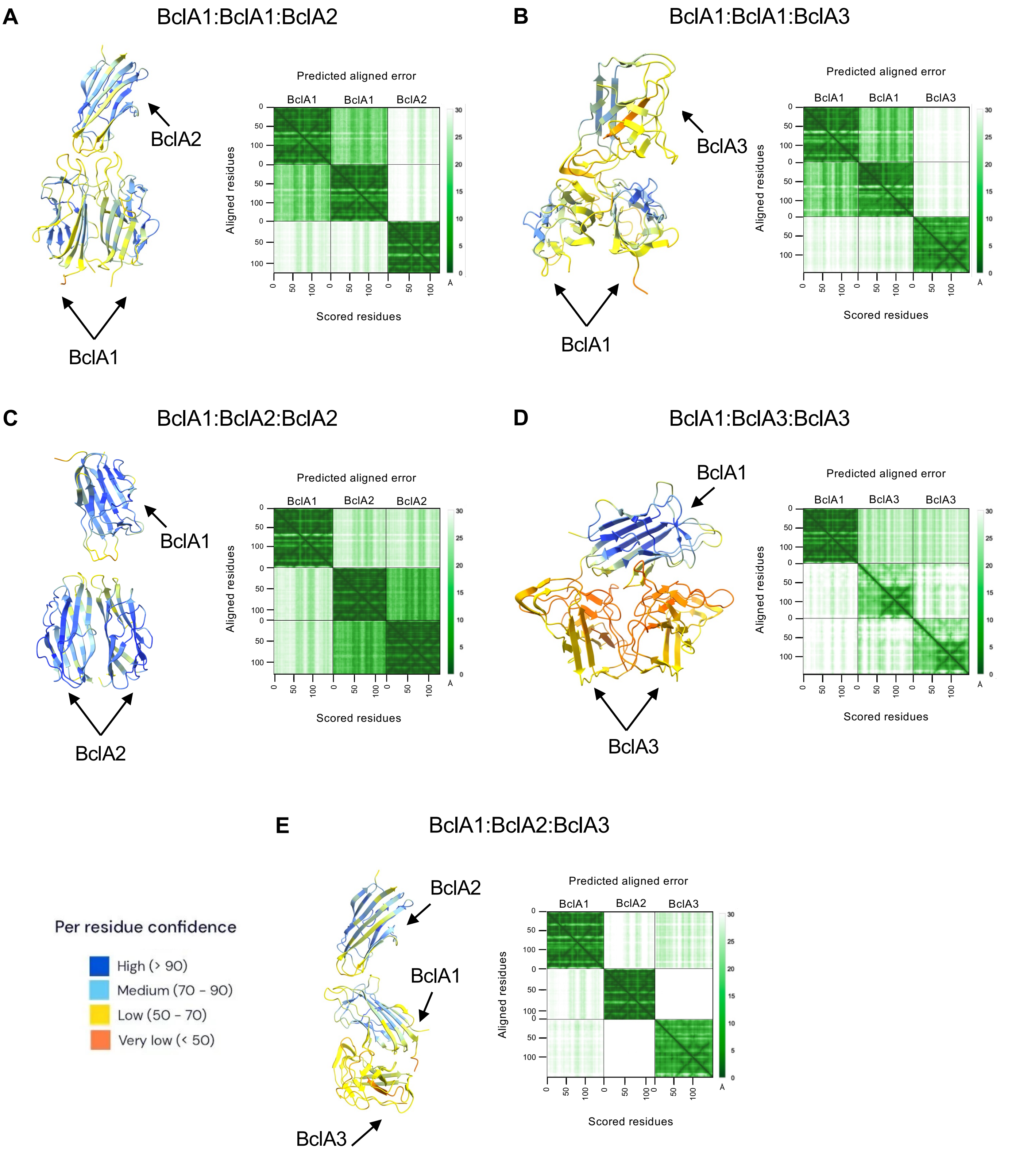

Fig. S10. AlphaFold3 predicted CTD heterotrimer structures from strain 630. Heterotrimer prediction composed of **(A)** 2 units of BclA1_CTD and 1 unit of BclA2_CTD, **(B)** 2 units of BclA1_CTD and 1 unit of BclA3_CTD, **(C)** 1 unit of BclA1_CTD and 2 unit of BclA2_CTD, **(D)** 1 unit of BclA1_CTD and 2 unit of BclA3_CTD or **(E)** 1 unit of BclA1_CTD, 1 unit of BclA2_CTD and 1 unit of BclA3_CTD. Each model has its respective Predicted aligned error (PAE) plot.

Figure S11

A

```
BclA1              1 SQPVANFLVNAPSPQTLNNGDAITGWQTIIGNSSSITVDTNG--------     42
                                   ...|:|    |....:|.:..:..:|.|
BclA               1 --------------YAFNSG----GISLDLGINDPVPFNTVGSQFGTAIS     32

BclA1             43 -----TFTVQENGVYYISVSVALQPGSSSINQYSFAILFPILGGKDLAGL     87
                          ||.:.|.|.|  .::|.....::|:                |.||
BclA              33 QLDADTFVISETGFY--KITVIANTATASV----------------LGGL     64

BclA1             88 TTE------PGGGGVLSGYFAGFLFGGTTFTINNFSSTTV---GIRNGQS    128
                     |.:      ||.|..|....|..:....|......|...|   |:....:
BclA              65 TIQVNGVPVPGTGSSLISLGAPIVIQAITQITTTPSLVEVIVTGLGLSLA    114

BclA1            129 AGTAATLTIFRIADTVMT    146
                     .||:|::.|.::|
BclA             115 LGTSASIIIEKVA-----    127
```

B

```
BclA2              1 ANNAQFTVSSS----SLGNNTLVTFN---SSFINGTNITFPTSSTINLAV     43
                          :..:|.    .||.|..|.||   |.|  ||.|:...:.|..::.
BclA               1 -----YAFNSGGISLDLGINDPVPFNTVGSQF--GTAISQLDADTFVISE     43

BclA2             44 GGIYNVS-FGIRAILSLAGFMSITTNFNGVAQNN-----------FIAKA     81
                     .|.|.:: ....|..|:.|  .:|...|||....           .:.:|
BclA              44 TGFYKITVIANTATASVLG--GLTIQVNGVPVPGTGSSLISLGAPIVIQA     91

BclA2             82 VNTLTSSDVSVSLSFLVDARAAAVTLSFTFGSGTTGTSPAGYVSVYRIQ    130
                     :..:|::      ..||:.....:.||...|:..:       :.:.::.
BclA              92 ITQITTT------PSLVEVIVTGLGLSLALGTSAS-------IIIEKVA    127
```

C

```
BclA3              1 -----SQPVANFLVNAPSPQTLNNGDAITGWQTIIG---NSSSITVDTNG     42
                          |......||||    .|.|    ||  |::|   :...|.....|
BclB               1 IIPFASGTTPALLVNA----VLAN----TG--TLLGFGFSQPGIAPGVGG     40

BclA3             43 TFTVQENGVYYISVSVALQPGSSSINQYSFAILFPILGGKDLAGLTTEPG     92
                     |.|:              .||  .:..|:|      :..:|  |:.|.
BclB              41 TLTI--------------LPG--VVGDYAF------VAPRD--GIITS--     64

BclA3             93 GGGVLSGYFAGFLFGGTTFTINNFSSTTVGIRNGQSAGTAATLTIFRIAD    142
                         |:|:|:.        |......|.|.|:.......||:.|...:|.
BclB              65 ----LAGFFSA--------TAALAPLTPVQIQMQIFIAPAASNTFTPVAP    102

BclA3            143 TVMT----------------------------------------------    146
                     .::.
BclB             103 PLLLTPALPAIAIGTTATGIQAYNVPVVAGDKILVYVSLTGASPIAAVAG    152

BclA3            147 ---------    146

BclB             153 FVSAGLNIV    161
```

D

Aligned sequence	Identity	Similarity
BclA1 - BclA	31/168 (18.5%)	51/168 (30.4%)
BclA2 - BclA	29/149 (19.5%)	53/149 (35.6%)
BclA3 - BclB	33/209 (15.8%)	50/209 (23.9%)

Fig. S11. Pairwise alignment (PSA) of *C. difficile* BclA_CTD with *B. anthracis* BclA/BclB_CTD. EMBOSS Needle was used for PSA of **(A)** Cd_BclA1_CTD$_{630}$ with Ba_BclA_CTD **(B)** Cd_BclA2_CTD$_{R20291}$ with Ba_BclA_CTD and **(C)** Cd_BclA3_CTD$_{R20291}$ with Ba_BclB_CTD. **(D)** Percentage of identity of aligned sequences. CTD: C-terminal domain. The genomes of *C. difficile* 630 (AM180355.1), *C. difficile* R20291 (CP029423), and *B. anthracis* (AE016879.1) were used as references.

Figure S12

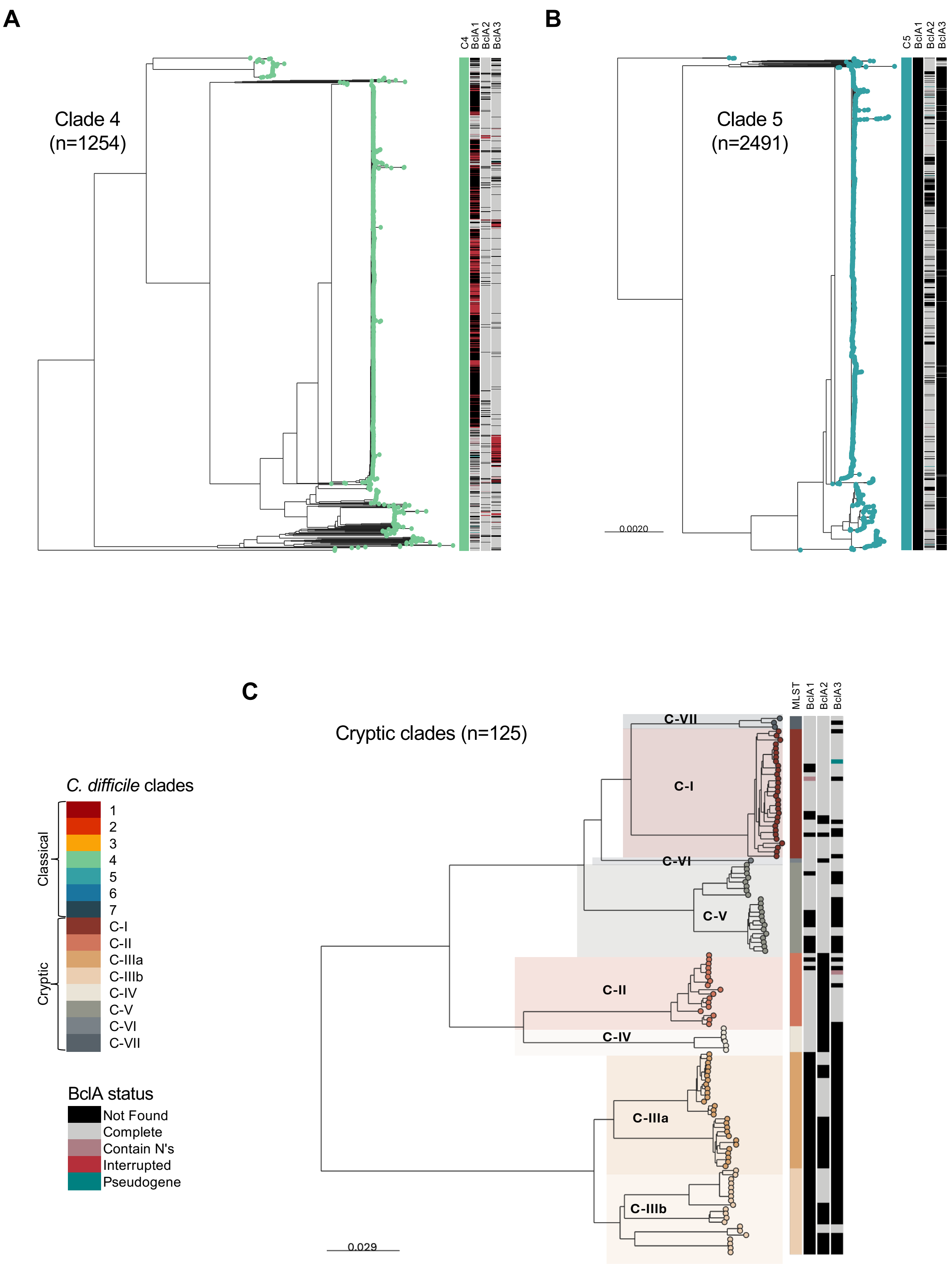
A
Clade 4
(n=1254)
C4
BclA1
BclA2
BclA3
B
Clade 5
(n=2491)
C5
BclA1
BclA2
BclA3
0.0020
C
Cryptic clades (n=125)
C-VII
C-I
C-VI
C-V
C-II
C-IV
C-IIIa
C-IIIb
MLST
BclA1
BclA2
BclA3
0.029
C. difficile clades
Classical
Cryptic
1
2
3
4
5
6
7
C-I
C-II
C-IIIa
C-IIIb
C-IV
C-V
C-VI
C-VII
BclA status
Not Found
Complete
Contain N's
Interrupted
Pseudogene

Figure S13

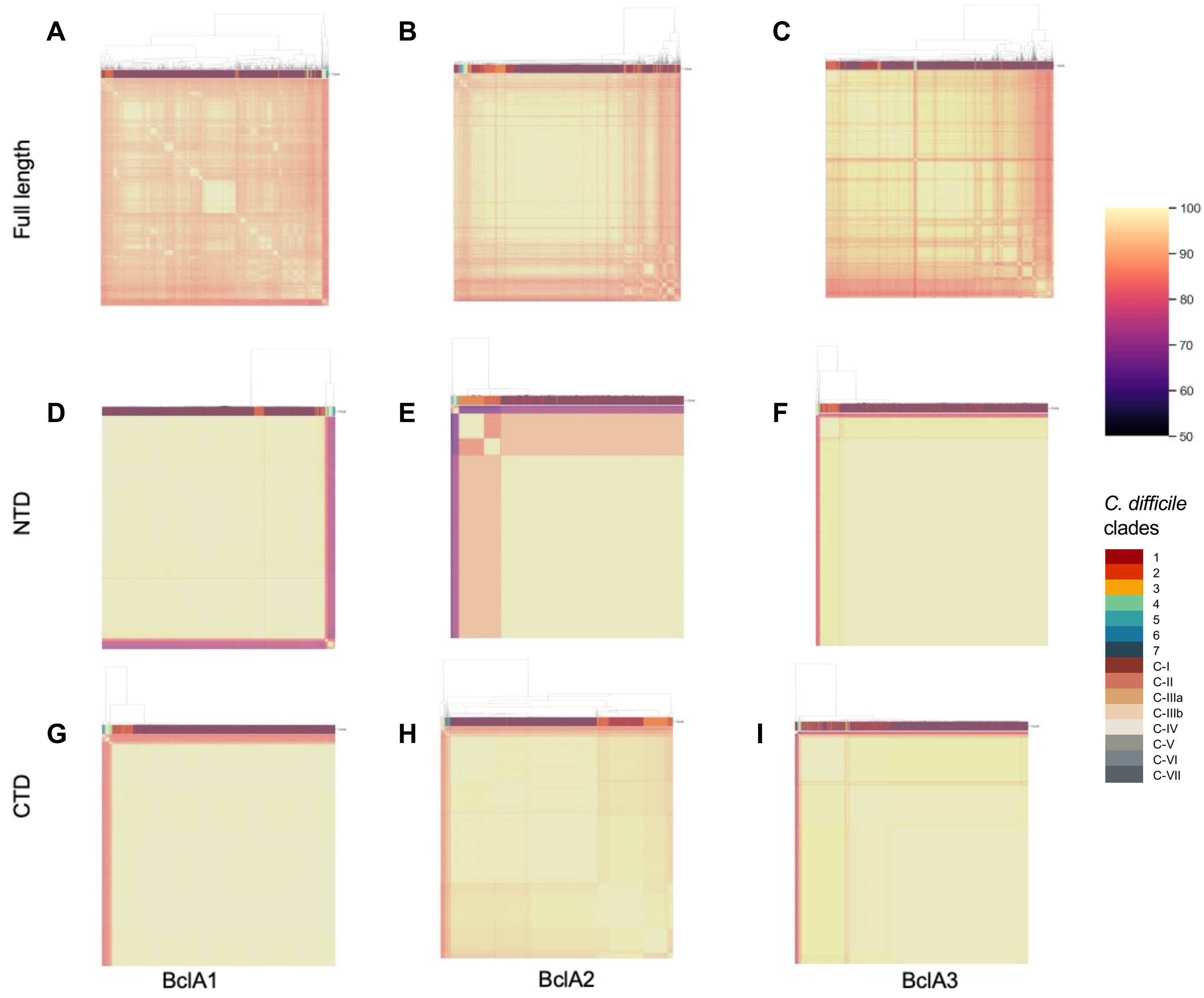

Fig. S13. Average amino acid identity (AAI) analysis of unique BclA. Heatmap of the percentage of identity between all unique **(A)** BclA1, **(B)** BclA2 and **(C)** BclA3. AAI was performed for full length protein, NTD and CTD regions. N-terminal domain (NTD) and C-terminal domain (CTD).

Figure S14

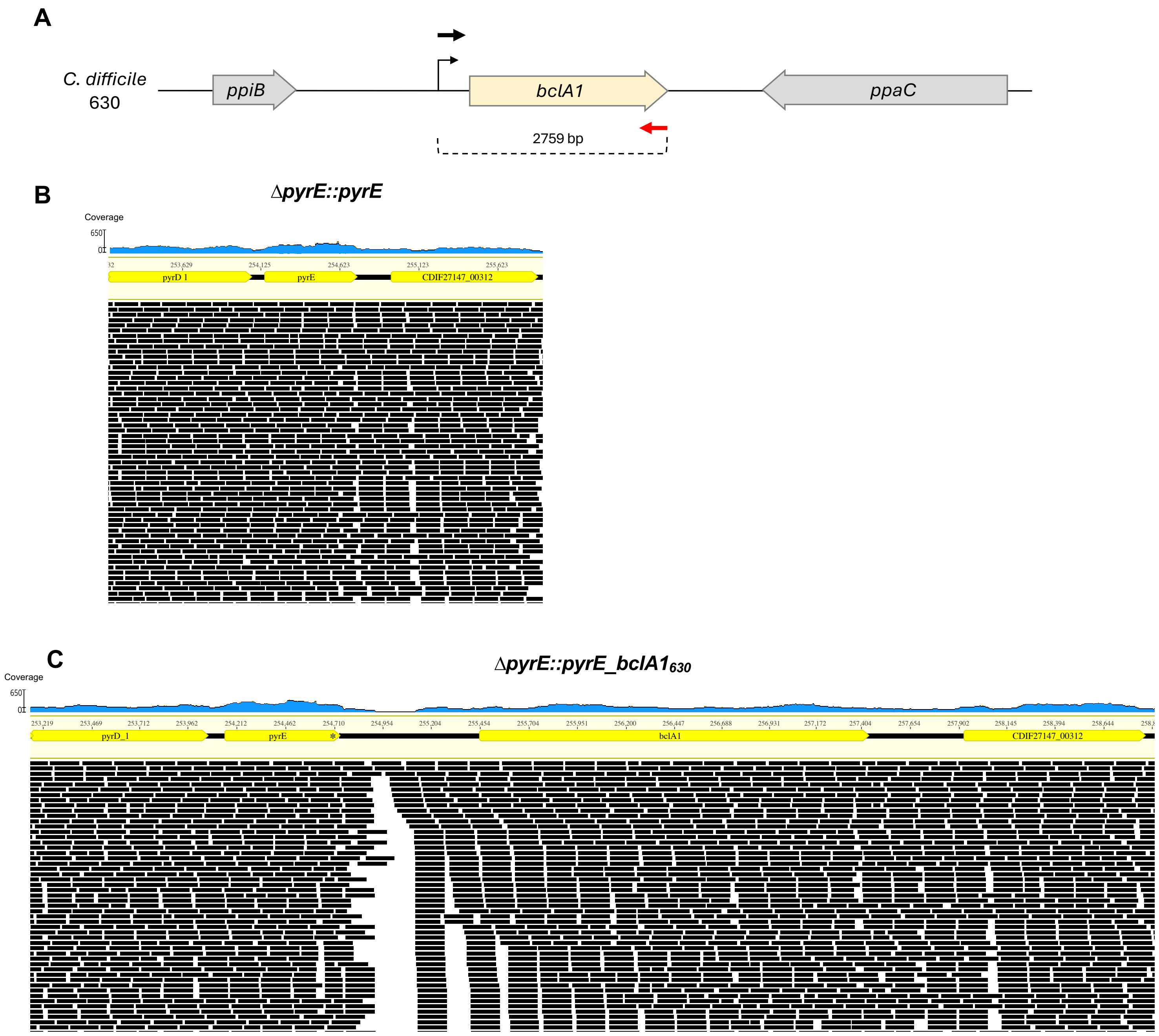

Fig. S14. Complementation of *bclA1$_{630}$* allele to *C. difficile* R20291 genome. (A) Genetic context of *bclA1$_{630}$*. The region amplified is composed of 677 bp upstream *bclA1* plus the complete *bclA1* ORF (2082 bp). The black and red arrows correspond to the forward and reverse primers. **(B)** Read mapping of *C. difficile* Δ*pyrE::pyrE* to *C. difficile* R20291 (CP029423) reference genome, zoom in to *pyrE* locus. **(C)** Read mapping of *C. difficile* Δ*pyrE::pyrE_bclA1$_{630}$* to *in silico* constructed genome, zoom in to *pyrE* locus.
